# HCN1 and HCN2 in Rat DRG Neurons: Levels in Nociceptors and Non-Nociceptors, NT3-Dependence and Influence of CFA-Induced Skin Inflammation on HCN2 and NT3 Expression

**DOI:** 10.1371/journal.pone.0050442

**Published:** 2012-12-07

**Authors:** Cristian Acosta, Simon McMullan, Laiche Djouhri, Linlin Gao, Roger Watkins, Carol Berry, Katherine Dempsey, Sally N. Lawson

**Affiliations:** 1 School of Physiology and Pharmacology, University of Bristol, Bristol, United Kingdom; 2 Australian School of Advanced Medicine, Macquarie University, Sydney, Australia; 3 Department of Biomedical Sciences, Faculty of Medicine, King Faisal University, Al-Ahssa, Saudi Arabia; 4 Department of Physiology, Tongji Medical School, Huazhong University of Science and Technology, Wuhan, China; Virginia Commonwealth University, United States of America

## Abstract

I_h_, which influences neuronal excitability, has recently been measured *in vivo* in sensory neuron subtypes in dorsal root ganglia (DRGs). However, expression levels of HCN (hyperpolarization-activated cyclic nucleotide-gated) channel proteins that underlie I_h_ were unknown. We therefore examined immunostaining of the most abundant isoforms in DRGs, HCN1 and HCN2 in these neuron subtypes. This immunostaining was cytoplasmic and membrane-associated (*ring*). Ring-staining for both isoforms was in neurofilament-rich A-fiber neurons, but not in small neurofilament-poor C-fiber neurons, although some C-neurons showed cytoplasmic HCN2 staining. We recorded intracellularly from DRG neurons *in vivo*, determined their sensory properties (nociceptive or low-threshold-mechanoreceptive, LTM) and conduction velocities (CVs). We then injected fluorescent dye enabling subsequent immunostaining. For each dye-injected neuron, ring- and cytoplasmic-immunointensities were determined relative to maximum ring-immunointensity. Both HCN1- and HCN2-ring-immunointensities were positively correlated with CV in both nociceptors and LTMs; they were high in Aβ-nociceptors and Aα/β-LTMs. High HCN1 and HCN2 levels in Aα/β-neurons may, via I_h_, influence normal non-painful (e.g. touch and proprioceptive) sensations as well as nociception and pain. HCN2-, not HCN1-, ring-intensities were higher in muscle spindle afferents (MSAs) than in all other neurons. The previously reported very high I_h_ in MSAs may relate to their very high HCN2. In normal C-nociceptors, low HCN1 and HCN2 were consistent with their low/undetectable I_h._ In some C-LTMs HCN2-intensities were higher than in C-nociceptors. Together, HCN1 and HCN2 expressions reflect previously reported I_h_ magnitudes and properties in neuronal subgroups, suggesting these isoforms underlie I_h_ in DRG neurons. Expression of both isoforms was NT3-dependent in cultured DRG neurons. HCN2-immunostaining in small neurons increased 1 day after cutaneous inflammation (CFA-induced) and recovered by 4 days. This could contribute to acute inflammatory pain. HCN2-immunostaining in large neurons decreased 4 days after CFA, when NT3 was decreased in the DRG. Thus HCN2-expression control differs between large and small neurons.

## Introduction

I_h_ regulates neuronal excitability [1] and has been implicated in pathological pain [2]–[5]. To understand the factors contributing to I_h_ and its properties in sensory neuronal subgroups, the levels of expression of channel proteins underlying I_h_ in these subgroups needed to be determined. These channel proteins are the hyperpolarisation-activated, cyclic nucleotide-gated (HCN) non-selective cation channels. There are four isoforms (HCN1-4) [6]–[8]. Previous studies have shown that mRNAs for HCN1 and HCN2 were the most abundant in rat DRGs, and also showed the greatest immunoreactivities in large DRG neurons, while HCN3 and HCN4 were very low or undetectable [2], [4]. HCN2 mRNA was later also reported in small DRG neurons [9].

Our recent paper [10], showed that: a) I_h_ was most prominent in Aα/β-neurons, less evident in Aδ-neurons and minimal in most C-neurons, confirming previous findings [11], [12]; b) the I_h_ magnitudes in nociceptors and low threshold mechanoreceptors (LTMs) were positively correlated with their conduction velocities (CVs); c) I_h_ was high in Aβ-nociceptors and cutaneous Aα/β LTMs but d) I_h_ was highest in muscle spindle afferents (MSAs), and was elevated in some C-LTMs above levels in C-nociceptors. Compared to heterologously expressed channels, I_h_ activation rates in many subgroups of DRG neurons *in vivo* were closest to those for HCN1 or fell between those of HCN1 and HCN2 [10] and were much shorter than those reported for HCN3 or HCN4 [13], suggesting that HCN1 and HCN2 underlie I_h_ in these neurons. However, despite the high I_h_ in many DRG neurons, and despite I_h_ having been implicated in chronic pain, there was no data on expression of HCN1 and HCN2 in DRG neurons with identified sensory properties (either nociceptors or LTMs).

However, comparisons between *in vivo* and *in vitro* HCN channel expressions and I_h_ in DRG neurons are hampered by lack of information about their dependence on the *in vivo* environment. Differences are suggested by low I_h_ densities (ranging from 1.2 to 5.3 pA/pF) in large neurons (>42 µm diameter) in >24 hr cultures e.g. [2], [14] compared with *in vivo* I_h_ densities for Aα/β-neurons (ranging from 20.4 to 35.2 pA/pF) [10]. An obvious candidate is neurotrophin-3 (NT3) because it: a) has important influences on large DRG neurons [15], [16], and b) has not been added to DRG culture media used for I_h_ studies.

Finally, emerging evidence for a role of HCN2-related I_h_ in inflammatory pain includes: a) the lack of HCN2 in HCN2−/− mice prevents acute (seconds/minutes) responses to inflammatory mediators in small DRG neurons [3] and b) a longer term role 5–7 days after chronic (CFA-induced) inflammation when HCN2 was upregulated *in vivo* in small DRG neurons; and because blocking I_h_ at these times reduced C-fibre spontaneous firing, there was a potential link between HCN2 and this firing [5]. However, HCN2 expression had not been examined 1 day after CFA when the spontaneous firing in intact C-neurons was greatest [17]. We therefore examined whether HCN2 expression was upregulated in DRG neurons at earlier times (i.e. 1–4 days) after this inflammation. Our finding that large neurons had lowered HCN2 expression 4 days after CFA, raised the question about whether NT3 decreased in the DRG at this time, which might account for this.

To answer these questions, we investigated the relationship of HCN1- and HCN2-immunoreactivity to sensory properties and CV in lumbar (L4-L6) DRG neurons, *in vivo,* in individual neurons by combining intracellular recording, sensory property identification and fluorescent-dye injection, followed by immunocytochemistry. We examined whether greater expression of HCN1 or HCN2 might explain the previously reported findings of a) the very high I_h_ in MSAs, or b) the elevated I_h_ in some C-LTMs compared to C-nociceptors. We also examined *in vitro* whether ring-immunostaining was dependent on NT3. We quantified HCN2-immunostaining 1 and 4 days after cutaneous CFA-induced inflammation and examined NT3 levels in DRGs (Western blots and immunocytochemistry).

## Materials and Methods

### Primary Antibodies

The anti-HCN1 and anti-HCN2 antibodies were rabbit polyclonal antibodies from Alomone Labs Ltd, Jerusalem, Israel. Anti-HCN1 was raised against peptides 6–24 of rat HCN1, anti-HCN2 against peptides147–161 of human HCN2. These antibodies had previously been characterized using channels expressed in COS-7 or HEK cells. Specificities for both these antibodies had been further confirmed by Western blots in dog Purkinje fibers and ventricular myocytes [18] and for HCN1 only in adult rat tissues including DRG [2]. However, because we found multiple bands with rat brain tissue (see Fig. S1), we undertook further characterisation with a) Western Blots using a variety of tissues for the anti-HCN1 and anti-HCN2 antibodies (for details see Fig. S1); b) antibody pre-absorption to examine whether these antibodies crossreact between these two isoforms (Fig. S1) and c) selective knockdown of HCN2 using siRNA to determine the specificity of this HCN2 antibody in the DRG (Fig. S2).

For double labelling studies, for neurons we used Isolectin B4 (IB4) conjugated with Alexa 488 which binds to small, C-nociceptor neurons in the DRG [19]. We also used RT97 (gift from JN Wood) a mouse monoclonal antibody against a highly phosphorylated form of the 200 kDa neurofilament (NF) subunit, that selectively labels DRG neuron somata with myelinated A-fibers [20], [21]. For double fluorescent staining of the nodes of Ranvier we used antibodies against HCN1 or HCN2, plus a mouse monoclonal antibody against Ankyrin G (Oncogene, clone 463, cat. # NB-20). This antibody has been used for immunohistochemistry and characterized by western blot e.g. [22] and is routinely used to label nodes of Ranvier e.g. [23]–[25]. NT3 expression was examined using a rabbit polyclonal antibody from Abcam (Cambridge, UK, ab65804) mapping a middle region of human NT3 conserved in mice and rat. This antibody has been characterized by the manufacturer by western blot and has also been used and characterized further in the literature [26], [27].

### *In vivo* Intracellular Recording

#### Ethics statement

All procedures were performed under a Home Office UK licence held under, and in strict accordance with, the provision of the UK Animals (Scientific Procedures) Act 1986; they had also been reviewed by the University of Bristol Ethical Review Group. All rats were killed at the end of experiments with an anaesthetic overdose.

#### Animal preparation

For further details of preparation for rats for electrophysiological recordings in DRG neurons see [19], [28]–[30]. 150–180 g female Wistar rats were deeply anaesthetized with sodium pentobarbital (70–80 mg/kg, i.p.) so that there was no withdrawal reflex in response to pinch of the forepaw in order to minimize suffering. Fur on the ipsilateral (left) hindlimb was clipped short. A tracheotomy enabled artificial ventilation and end-tidal CO_2_ monitoring. Cannulations of the right carotid artery and external jugular vein enabled, respectively, blood pressure monitoring and i.v. injections. Throughout the experiment additional anaesthetic (10 mg/kg, i.v.) was administered hourly; we have shown this dose maintains deep anaesthesia throughout [28]. The left L4-L6 DRGs and dorsal roots were exposed. The liquid paraffin pool (dental impression paste, Xantopren VL plus, Hanau, Germany) was maintained at ∼30°C (28.5∼32°C); core temperature was 35±0.5°C.

Recording stability was improved by raising the DRG slightly by inserting a small silver platform prior to recording. For stimulating the DRG neurons, the dorsal root was cut near the spinal cord and draped within the paraffin pool over a pair of bipolar platinum electrodes. The left hindpaw was fixed with the plantar surface exposed upwards for sensory testing and the dorsal surface was fixed (downwards) with superglue. This also improved stability during receptive field testing. Blood pressure was ∼80–100 mm Hg, and was stable throughout, indicative of deep anaesthesia. Muscle relaxant (pancuronium bromide, 1 mg/kg i.v.) was administered before recording, and again at approximately hourly intervals, in all cases accompanied by additional anaesthetic (see above).

#### Intracellular recordings

Borosilicate microelectrodes were filled with a fluorescent dye: Lucifer yellow (LY: 5 mg/ml in 0.1 M LiCl; 200–500 MΩ tip resistance), ethidium bromide (EB: 6 mg/ml in 1 M KCl; 80–120 MΩ), or cascade blue (CB: 30 mg/ml in 0.1 M LiCl; 260–600 MΩ). The microelectrode was advanced in 1 µm steps. When a stable Em was recorded, a rectangular stimulating pulse (0.03 ms duration for A-fiber neurons or 0.3 ms for C-fiber neurons) was applied to the dorsal root. The intensity was increased gradually until a somatic action potential (AP) was evoked. Records were amplified (Axoclamp 200A), and digitized (1401plus, at 40 KHz, Cambridge Electronic Design, UK) and recorded by a PC running CED Spike 2 versions 4–6.

#### Conduction velocity (CV)

CV was calculated from the conduction distance between cathode and the recorded neuron soma, divided by latency (including utilization time) to the initiation of the somatic AP, see [31]. CVs were classified as: C (<1.0 m/s), Aδ (1.5∼6.5 m/s) or Aα/β (>6.5 m/s) using boundaries measured from dorsal root compound action potentials under the same conditions (temperature, gender, rat weight) [29].

#### Sensory receptive properties

Hand-held stimulators applied to the hind limb and flank were used to determine sensory properties for the DRG neuron being recorded, see [32]–[34] for further details. Firstly, light touch, brushing, tapping, stretching, and light pressure were applied; neurons were low-threshold mechanoreceptors (**LTMs**) if they responded to these stimuli.

In all CV groups there were LTMs and nociceptors. The LTMs were classed as follows:

Aα/β-neurons included muscle spindle afferents (**MSA**s), cutaneous LTMs and Aβ-nociceptors. MSAs responded to gentle pressure and vibration (100–250 Hz), their receptive fields did not move if the overlying skin was moved; many had ongoing firing due to a) muscle stretch from hind limb extension and b) the muscle relaxant, see [34]. Cutaneous Aα/β-LTMs included a) rapidly adapting (**RA**) units that responded best to moving stimuli: in glabrous skin they were glabrous RA and in hairy skin these were guard hair (**G**) or field (**F**) units; and b) slowly adapting (**SA**) units with sustained firing in response to sustained gentle pressure. Aδ-LTM units were D hair units with large receptive fields that responded to slow movement of hair, cooling and skin stretch. C-LTMs responded to very slow movement across skin and to cooling.

If there were no responses to non-noxious stimuli, noxious mechanical stimuli were applied, initially moderate pressure, then needle prick, fine pinch or squeeze (flat or toothed forceps). To test for responses to cooling, we used a localized very brief spray of ethylchloride. Heat responses of mechanical nociceptors with superficial receptive fields were tested with hot water (>50°C). A-fiber nociceptors were high-threshold mechanoreceptors (**HTMs**) with superficial or deep (subcutaneous) receptive fields. Aβ-nociceptors included moderate pressure nociceptors that responded modestly to moderate pressure, but gave greater responses to noxious pressure or pinch [35]. C-nociceptors included HTMs with superficial or deep receptive fields. Those with superficial receptive fields included mechano-heat [36] and mechano-cold units. Note that we cannot exclude the possibility that nociceptors with deep receptive fields might have responses to thermal stimuli as these were not tested due to thermal insulation properties of epidermis see [37]. Units that responded only to cooling were called cooling units.

Neurons classed as unresponsive did not respond to any of the mechanical stimuli above, either because we did not find their receptive field or because they were very high threshold “silent” nociceptors. Unresponsive C-neurons with APs that were typical of nociceptors, with long afterhyperpolarisations [30], [38], were called “nociceptor-type”. They were included on graphs with nociceptors but are indicated with star symbols. We included no unresponsive A-fiber neurons.

#### Neuronal dye-labeling

After recording somatic APs and identification of sensory receptive properties, neurons were injected with fluorescent dye. Detailed methods for dye-injection into identified DRG somata have been described previously [28], [29], [33], [34]. Dye injections were made with 500 ms square wave pulses of positive current for EB or negative current for LY or CB (±1.3 nA) at 1 Hz for up to 10 min for A-fiber neurons and up to 5 min for C-fiber neurons. After dye injection, sensory properties and CV were again checked to ensure that the electrode tip remained in the same neuron.

After perfusion-fixation under terminal deep anaesthesia (see below), DRGs were removed and post-fixed (below) and conduction distances were measured. Serial frozen sections were then cut (described below). Each section was scanned for dye-injected neurons under epi-fluorescence on a Leica DMRBE microscope (Wetzlar, Germany) and photomicrographs of dye-injected neurons were captured (40× objective) using a Hamamatsu digital camera. Dye-labelled neurons were rejected if they were only very weakly fluorescent, if position and/or depth in the DRG differed from records made during the dye-injection, or if more dye-labelled neurons than expected were found [33].

### Tissue Preparation for Immunocytochemistry

Female rats (150 to 180 g) under deep terminal anaesthesia (i.p. pentobarbital 60–80 mg/kg) were perfused transcardially with 0.9% saline followed by Zamboni’s fixative [39]. DRGs and dorsal roots, both those from electrophysiologically recorded rats and from non-recorded rats, were removed and post-fixed for 60 mins in Zamboni’s solution and stored overnight at 4°C in 30% sucrose. Serial 7 µm cryostat sections were cut transversely through the entire length of the DRG as described previously e.g. [33]. Dorsal roots were cut longitudinally in serial 5 µm cryostat sections. Sections were placed on gelatine coated slides and were stored at −20°C until immunocytochemistry was performed.

### Immunocytochemistry

#### Avidin-biotin complex (ABC) immunocytochemistry

ABC staining of DRGs including the studies of dye-injected neurons was preceded by blocking of endogenous peroxidase and biotin-like activity with 3% H_2_O_2_ and an avidin–biotin blocking kit (Vector Laboratories, Peterborough, UK), respectively. Sections were then incubated for 30 mins with 10% normal goat serum [40] in PBS. ABC immunocytochemistry was performed with an ABC kit (Vector Laboratories). Briefly, sections were incubated overnight at 4°C in primary antibody (1∶1000 for HCN1; 1∶2000 for HCN2; 1∶1000 for NT3) in 0.05% Triton X-100 in PBS buffer with 1% NGS. After washes, sections were incubated for 30 mins with biotinylated anti-rabbit IgG (1∶200; Vector Laboratories). 3, 3′-diaminobenzidine (DAB) was used to form a coloured reaction product. No staining resulted if the primary antibody was omitted. Immunocytochemistry for all tissues used in comparisons of normal and CFA treated DRG was simultaneous, with identical times/temperatures at all stages.

#### Immunofluorescence

For double immunofluorescence staining of DRG or dorsal root nerve sections from 2 rats were rehydrated with PBS for 10 min and then permeabilized 5 min with 0.2% v/v triton-X100 and blocked for 1 hr at room temperature with 5% BSA+10% foetal calf serum. Anti-HCN1 (1∶1000) and anti-HCN2 (1∶2000) staining was performed overnight at 4°C followed by rinsing with PBS and incubation for 1 hr at room temperature with a donkey anti-rabbit Alexa 594 antibody (1∶400, Molecular Probes). After rinsing off the secondary antibody with PBS, sections were incubated overnight at 4°C with either mouse anti-NF200 (RT97, 1∶4000) or Ankyrin G (1∶200). On the next day, sections were incubated for 1 hr at room temperature with a goat anti-mouse 488 secondary antibody (1∶400, Molecular Probes), then washed and mounted.

### Image Analysis

#### Dye-injected in vivo-recorded neurons

Images of immunocytochemical reaction product were captured under bright-field optics (40× objective). Ring staining over the soma membrane (see Results) and cytoplasmic staining were examined for sections of dye-injected neurons that were immunostained for either HCN1, or HCN2. With the observer blind to the receptor type, ring immunointensity was scored subjectively: 0 = negative, 1 = just visible and 5 = equivalent intensity to the most intense ring staining for that antibody in that section.

As a separate procedure, the relative intensities of ring and cytoplasmic immunostaining for HCN1 and HCN2 separately were determined semi-quantitatively, using previously described methods [29], modified as follows for analysis of relative ring intensity. The following measures were taken of immunointensity for each dye-injected neuron: 1) the mean ring pixel density (designated ***r***) was the average pixel density under three lines drawn within the thickness of the ring, avoiding regions that were cut obliquely and were thus wider and paler than the rest of the ring; 2) the average cytoplasmic pixel density (designated ***c***) over the whole cytoplasm excluding both the nucleus and the ring or outermost region of the cytoplasm if no ring was visible. Then within the same section as the dye-injected neuron, the minimum neuronal cytoplasmic staining (designated ***a***) was determined as the average cytoplasmic pixel density of the three least stained neurones, and the maximum ring staining was the average ring pixel density of the three neurons with densest ring staining) (***b***, 100%). For the dye-injected neuron, percentage relative intensity of ring and cytoplasmic staining were obtained as follows: Relative intensity = 100×((***r*** or ***c***)−***a***)/(***b***−***a***). From this point, the terms *ring intensity* and *cytoplasmic intensity* are used to indicate relative intensities as determined above, in contrast to *subjective scores*.

#### HCN1 and HCN2 median ring intensities versus published I_h_ medians

For each of the sensory subgroups, we plotted the median (relative intensity) values for both HCN1 and HCN2 against previously determined median I_h_ values that we published previously (Table 1 in [10]). Briefly, in that study I_h_ was determined with discontinous single electrode voltage clamp in DRG neurons *in vivo* (for full Methods see [10]). The dye-filled electrodes of the present study were not suitable for I_h_ recording, and thus I_h_ and HCN could not be studied in the same neurons. However, since the sensory subgroups in the I_h_ paper had been defined using the same methods as in the present study, the groups were comparable.

### DRG Cell Cultures

Cultures were used for a) antibody characterisation (see Figs. S1 and S2) and b) for determination of the effect of NT3 on HCN1 and HCN2 immunostaining. DRG from all rostrocaudal levels were dissected from terminally anaesthetised (60–80 mg/kg pentobarbitone i.p.) female Wistar rats of 160–180 g. Neurons were cultured following previously published protocols [41]. Briefly, DRGs were, enzymatically digested (0.25% trypsin plus 0.5% collagenase for 45 min at 37°C) then mechanically dissociated; plated onto round coverslips coated with 10 ng/mm^2^ poly-D-Lysine plus 1 ng/mm^2^ laminin and kept at 37°C and 5% CO_2_ in Dulbecco’s MEM supplemented with N2 medium until used. Cell culture density at plating was ∼3×10^5^ neurons/mL. 10 µM β-D-cytosinearabinoside was used to control non-neural cell proliferation.

The dissociation and culture process is equivalent to acute axotomy, causes deprivation of normally available target- and nerve-derived trophic factors. Trophic factor supplements are therefore required to maintain a relatively normal phenotype *in vitro*. All neurons were cultured in the continuous presence of either mouse NGF 7S (10 ng/ml) or NGF 7S plus human recombinant NT3 (40 ng/ml). To examine the effect of NT3 on the immunostaining, cultures with NGF plus NT3 (NT3+) were compared with those treated only with NGF (NT3−). Both trophic factors were dissolved in the same medium (N2, see above). Culture medium supplemented with trophic factors was replaced daily.

#### Immunofluorescence on cultured neurons

Coverslips with cultured neurons growing on them were removed from the incubator at 1, 2 or 3 days *in vitro*, washed with PBS at 37°C for 5 min, then fixed in a solution of 4% w/v paraformaldehyde and 4.2%w/v sucrose in PBS (pH 7.2) for 10 min at room temperature, washed again in PBS and kept in PBS at 4°C overnight. Immunocytochemistry was performed the next day for NT3+ and NT3− cultures simultaneously. Cells were permeabilized for 4 min with 0.2% v/v Triton X-100 in PBS, blocked for 2–3 hr at room temperature with 5% bovine serum albumin +10% foetal calf serum and then incubated overnight at 4°C with either anti-HCN1 (1∶500) or anti-HCN2 (1∶1000) diluted in the blocking solution. Next day, coverslips were washed with PBS and incubated for 1 hr at room temperature with 1∶400 donkey anti-rabbit Alexa 594 (Molecular Probes, Invitrogen), washed again and mounted with FluorSave (Calbiochem, CA).

#### Image analysis in cultured neurons

For each treatment, in 12 cultures from 2 rats, images of 16–18 fields of 3.75×10^4^ µm^2^ were captured at 40X magnification under both interference contrast and fluorescence illumination with a Leica DMRBE microscope. Any neurons that appeared overly granular or with blebs under interference contrast were deemed unhealthy and excluded from analysis; all neurons that appeared healthy i.e. with a clear outline were analysed from fluorescence images as follows: cell area and staining intensity under a line drawn over the ring staining where that existed, and when a ring was not visible, over the perimeter of the neuron. Percentage relative intensities for all measured neurons, including those with and without NT3, were calculated with the method described for dye-injected neurons, using a range from 0% (minimum cytoplasmic staining with no NT3) to 100% (maximum ring staining with NT3).

### Studies of Effects of Cutaneous Inflammation Induced by CFA

Two 100 µl intradermal injections of Complete Freund’s Adjuvant (CFA) (Sigma, St. Louis, MO) were applied, under isoflurane anesthesia, to induce cutaneous inflammation. One injection was given into the mid-plantar surface of the hindpaw and the other lateral to the left knee as previously described [42]. Female Wistar rats (150–180 g) were four 1 day CFA and four 4 day CFA and three untreated rats. All rats were perfused under terminal anesthesia (see earlier).

#### Western Blots

These were carried out on pooled L4 and L5 tissue (i.e. 2 DRGs per pool) on ipsilateral, contralateral and untreated DRGs from 3 rats per treatment group (untreated, 1 day CFA and 4 days CFA). Western blots and protein extraction were performed as for HCN1 and HCN2 (for details see Methods S1). The rabbit anti-NT3 antibody was used 1∶1000 and the PVDF membranes were incubated overnight at 4°C. A semiquantitative analysis of the bands was carried out by drawing around the band in each lane to measure the mean pixel intensity. The score was the pixel density times the region of staining. This was done for the NT3 and the corresponding α-tubulin, then the ratio NT3:α-tubulin was determined for each lane.

#### Tissue preparation and immunocytochemistry

DRGs were removed from Zamboni’s perfused rats as for electrophysiological studies above. DRGs were post-fixed for 20 mins with Zambonis fixative, and series of 7 µms transverse sections taken at 400 µm intervals through the DRG were made. ABC immunocytochemistry was carried out simultaneously on series of sections from all rats for HCN2 and similarly for NT3.

### Image Analysis After CFA

One section through the middle of each L4 DRG was analysed from each rat i.e. unilaterally from the 3 untreated control rats, and ipsilaterally and contralaterally (separately) from each of four 1 day CFA rats and four 4 day CFA rats.

#### HCN2

All neurons with a visible nucleus in the section were analysed. Cytoplasmic staining level was determined by drawing around the cell outline and then around the nucleus and the mean (average) pixel density of the cytoplasm minus nucleus was determined. Then a line was drawn around the neuron, just inside the neuron perimeter, over the dense ring of staining if one was visible, and the mean (average) pixel density under that line was measured (edge intensity). The total cell cross-sectional area was also measured. In all cases 5–6 images (3.75×10^4^ µm^2^ each) per mid-section were captured at 40× magnification. The total number of neurons measured was: 175 for untreated rats, 247 and 309 for ipsilateral 1 day CFA and 4 day CFA treatments respectively.

#### NT3

For each L4 section, from images captured with the x20 objective, the mean pixel density for each of 9 representative neuron-rich/fiber poor areas (100×100 µm^2^ each, i.e.not single neurons as for HCN2) were determined with Image J. From these 9 values the median pixel density for each rat was determined.

### Statistics

All statistical comparisons of subgroups of neurons were non-parametric due to small numbers in groups, or groups failing the normality test. Thus comparison of medians in the scatter plots was with Kruskal-Wallis for three or more groups (solid lines indicate groups tested), with Dunn’s post hoc tests between all combinations of groups and with Mann Whitney tests for comparison of medians of two groups as stated in the figure legends. Correlations were with: linear regression analysis (regression lines and r^2^ values on graphs where significant) or the non-parametric Spearman correlations (Spearman’s correlation coefficient, r_s_, given where significant). All tests were performed with Prism 5 (GraphPad software, San Diego, CA). Tests were 2-tailed and a level of *p*<0.05 was considered statistically significant. Significance is indicated on all graphs by *p<0.05, **p≤0.01, ***p≤0.001, ****p≤0.0001.

## Results

**Figure 1:**
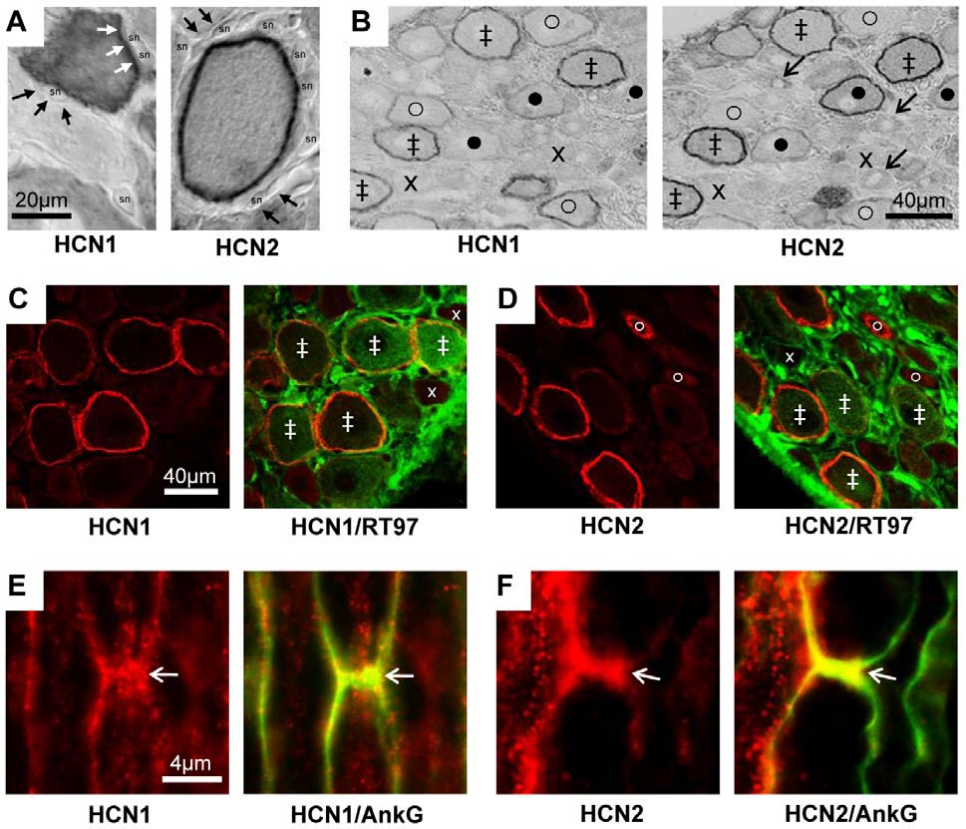
HCN1 and HCN2 immunostaining. **A and B**: ABC immunostaining. **A:** with X100 objective, HCN1 and HCN2 ring staining (white arrows) is over the neuron perimeter and not in satellite cells (black arrows) thus probably membrane associated, (sn = satellite cell nucleus). **B:** in adjacent sections HCN1 and HCN2 show co-localisation, with some differences in intensity or location. Symbols in **B**: ‡ ring staining for both HCN1 and HCN2; x neither, o clear HCN1 ring but weaker HCN2; • clear HCN2 ring but weaker HCN1. **C–F:** Double immunofluorescence staining in L5 DRG neurons. **C and D**: HCN1 (C) or HCN2 (D) ring staining (red) are present in large, myelinated, neurofilament-rich (RT97 positive, green) neurons. There is also cytoplasmic HCN2 staining in a few RT97 negative, unmyelinated, small neurons (fine arrow in D). Symbols in C and D indicate examples of staining with: ‡ both antibodies, x neither, o clear HCN2 cytoplasmic but not neurofilament staining. **E and F:** Representative X100 images of HCN1 (E) and HCN2 (F) (in red) with Ankyrin G (AnkG, green) to indicate the nodes of Ranvier (arrows) in longitudinal sections of normal L5 dorsal root nerve. Yellow is indicative of co-localisation.

Antibody characterisation shows specificity of both antibodies against their targets in DRGs by a) Western blots; b) pre-absorptions with blocking peptides (Fig. S1) and c) siRNA knockdown of HCN2 which highly significantly decreased HCN2 ring staining (Fig. S2). That these two antibodies distinguish clearly between the two isoforms, is shown in Fig. 1 and Fig. S1 C–F.

### HCN1 and HCN2 Immunostaining

In large sensory neurons there was some weak cytoplasmic staining but the most intense staining was in the form of a clear ring (Fig. 1) over the soma perimeter, as previously reported e.g. [2], [43], [44]. Additionally some small neurons showed staining; this was exclusively cytoplasmic (Fig. 1).

The ring/perimeter staining for both HCN1 and HCN2 was associated with the neuronal surface membrane, not with satellite cells (Fig. 1A). Some neurons, including the small neurons, had no visible ring (Fig. 1A, C and D, and Fig. S3). Despite similarities in ring staining for HCN1 and HCN2, some neurons were more strongly stained for HCN1 and others for HCN2 (Fig. 1B).

The clear ring-immunostaining for HCN1 and HCN2 was in medium and large RT97 positive (i.e. A-fiber [21] ) neurons but not in RT97 negative small (C-fiber) neurons (see Fig. 1C and D and Fig. S3). In myelinated sensory fibers in dorsal roots, the HCN1 and HCN2 immunostaining (red) is concentrated at nodes of Ranvier, identified by Ankyrin G (green) immunostaining (Fig. 1E and F).

### HCN1 Immunoreactivity in Dye-injected Normal DRG Neurons

HCN1 immunocytochemistry was successfully carried out for 74 neurons, from 50 rats. These neurons were injected with Lucifer yellow (LY, n = 45), ethidium bromide (EB, n = 26), or cascade blue (CB, n = 3).

**Figure 2:**
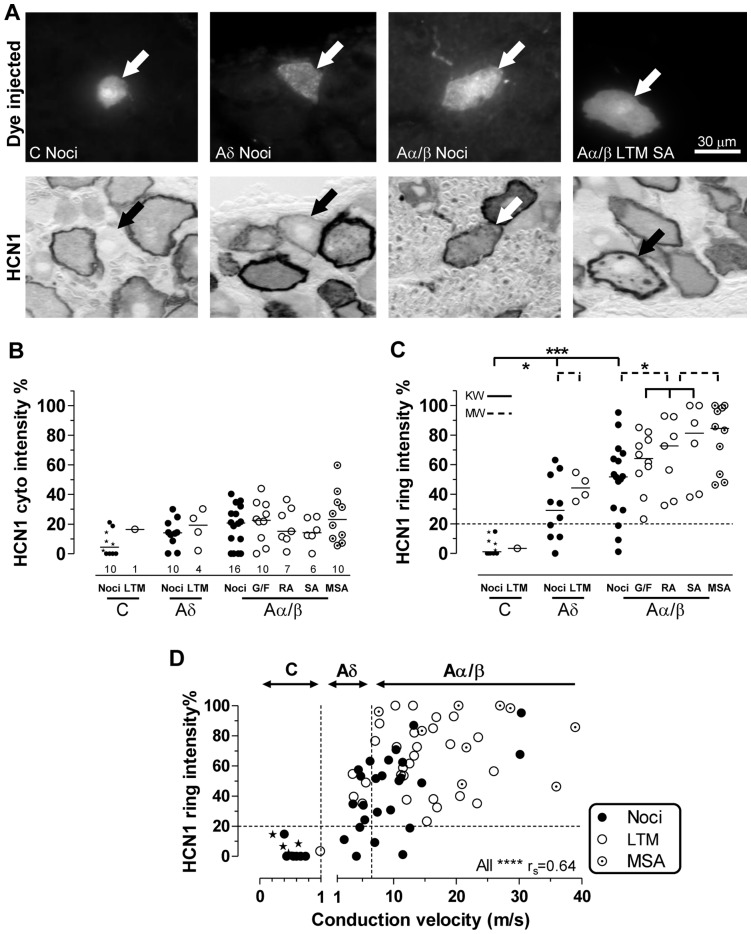
HCN1 immunointensity, sensory properties and CV. Images captured with 40X objective. **A:** From the left, a C-nociceptor, an Aδ-nociceptor, an Aα/β-nociceptor and an Aα/β-LTM SA unit all indicated by arrows. Top: fluorescent dye-injected neurons; bottom: bright field images of the same neurons after HCN1 immunocytochemistry (ABC). B–D: Relative intensities (see Methods) of HCN1 ring and cytoplasmic staining as a percentage of maximum ring intensity are plotted. **B** and **C:** scatter plots of HCN1 relative immunostaining intensity for different sensory properties. **B:** cytoplasmic staining, **C:** membrane-associated (ring) staining; above 20% (dotted line) ring staining is usually visible. Each symbol represents a single dye-injected neuron. Abbreviations: Noci: nociceptors; LTM: low threshold mechanoreceptor; G/F G hair or field unit; SA: slowly adapting; RA: rapidly adapting; MSA: muscle spindle afferent. Star symbols indicate C− and Aδ-nociceptor-type neurons. Tests were Kruskall-Wallis (solid lines) for comparison of 3 or more groups, or Mann-Whitney tests (for two groups, dotted lines). For more detail and significance levels see Methods. **D:** HCN1 ring relative intensity versus CV; vertical dotted lines indicate borderlines between C−, Aδ-, and Aα/β-fiber CVs. HCN1 ring intensities were highly correlated with CV in nociceptors (Spearman’s correlation, r_s_ = 0.7, p<0.0001) but not with LTMs.

Examples of fluorescent dye-injected DRG neurons with identified sensory properties are shown in Fig. 2A before (upper row) and after (lower row) HCN1-immunostaining. The C-nociceptor (typically) had no ring, the Aδ-nociceptor had a weak ring and the Aα/β-nociceptor and Aα/β-LTM SA unit both showed clear dark rings and some cytoplasmic staining.

Scatterplots of HCN1 cytoplasmic and ring staining intensities are shown in Fig. 2B and C. For numbers of units see Fig. 2B. The 10 C-nociceptor neurons include 6 C-nociceptors (solid dots) and 4 C-unresponsive nociceptors-type neurons (star symbols). The subjective scores for ring staining (≥1 for clearly visible rings), were highly correlated with objective HCN1 ring intensities for all neurons (P<0.0001, r^2^ = 0.87, not shown), demonstrating that the quantitative image analysis correlates well with the perceived intensity levels. All clearly visible rings had ring/edge staining intensities of ≥20% maximum ring staining (i.e. above the dotted line in Fig. 2 C and D).

#### HCN1 cytoplasmic intensities (Fig. 2B)

There was little or no cytoplasmic HCN1 immunostaining in most C-neurons. The median for the 10 C-nociceptors neurons was 4.5%, which was significantly lower than the median (17.6%) for all other neurons together (not shown, P<0.01, Mann Whitney test). For these other neuronal groups, all medians were <25%, and showed no significant differences between groups.

#### HCN1 ring intensities (Fig. 2C)

Neurons with clearly visible rings were most A-fiber LTMs, all Aβ-nociceptors, ∼75% of Aδ-nociceptors, no C-nociceptors and 0/1 C-LTMs.

Ring staining (including edge staining where no visible ring) differed between sensory neuron subgroups. C-neurons had the lowest edge intensities (median 1.2%, Fig. 2C) significantly lower than for all other neurons. Medians for Aδ-neurons were intermediate (35%), while those for Aα/β-neurons were the highest (64%). Ring intensities of Aα/β-neurons were significantly higher than both Aδ- and C-neurons (P<0.01 and P<0.001, respectively, not shown). The ring/edge intensities for all neurons were thus in descending order, Aα/β>Aδ>C. This order was also the same for nociceptors with Aβs having significantly more intense rings than Aδ- (p<0.01) and C-nociceptors (p<0.0001). It was also the case for LTMs, with Aα/β-LTMs having more intense HCN1-rings than Aδ-LTMs (p<0.05) and more than the single C-LTM. The Aα/β-LTMs together had the highest median ring intensities of all groups of neurons. The HCN1 ring staining was not significantly higher in MSAs than in other Aα/β-neurons.

The HCN1 ring/edge staining intensity was strongly correlated with CV (Fig. 2D) for all neurons, and for all nociceptors (P<0.0001, r_s_ = 0.7), but not for LTMs, perhaps due to too few data points for slowly conducting LTMs. Ring staining intensity was linearly correlated with cytoplasmic staining (not shown) for all neurons (P<0.0001, r^2^ = 0.55), for nociceptors (P<0.0001, r^2^ = 0.67) and for LTMs (P<0.01, r^2^ = 0.38). A possible interpretation is that cytoplasmic vesicles contain the channel accounting for cytoplasmic staining. These vesicles may become concentrated towards the edge of the cell contributing, in addition to the channel in the soma membrane, to the visible ring. Thus the cytoplasm may be a (less concentrated) reservoir of vesicular channel protein.

### HCN2 Immunoreactivity in Dye-injected Normal DRG Neurons

HCN2 immunocytochemistry was carried out successfully on 43 dye-injected neurons, 37 of which also had HCN1 immunocytochemistry carried out on an adjacent section. These were injected with LY (29), EB (13) or CB (1).

**Figure 3:**
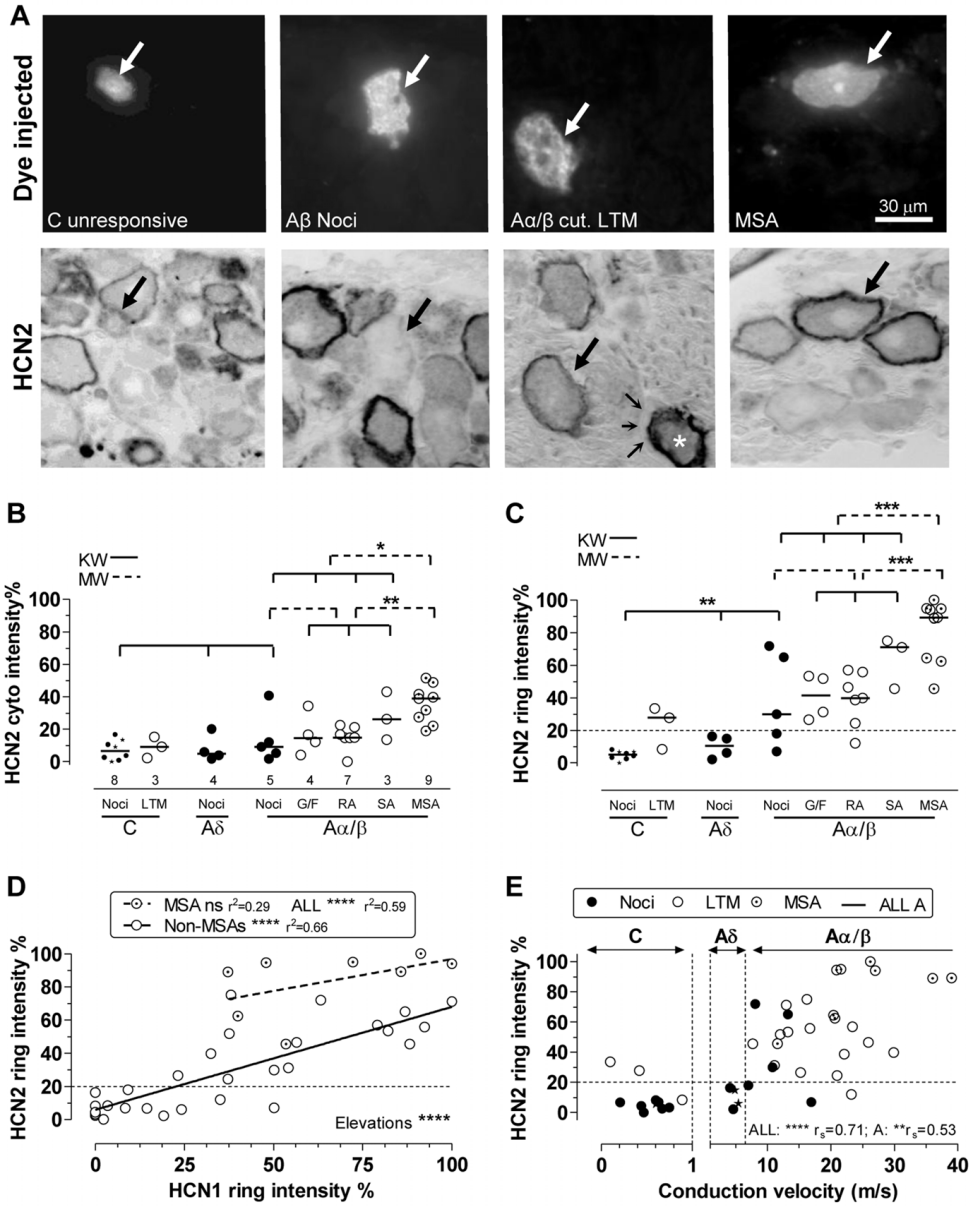
HCN2 immunointensity versus sensory properties and CV. **A**:Top row dye-injected neurons; bottom row; the same neurons after ABC HCN2 immunocytochemistry; all with X40 objective. Large arrows indicate the dye-injected neurons. From left to right: C-unresponsive neuron shows cytoplasmic but no ring staining; the Aβ-nociceptor illustrated shows no, or a very weak, ring; Aα/β cutaneous LTM (field unit) with clear, not intense, ring; in the same field the asterisk shows a (non-dye-labelled) neuron identified as an MSA by strong staining on an adjacent section (not shown) for the α3 Na^+^ pump, which selectively labels MSAs. Also in that image, fine arrows show satellite cells unstained by HCN2. The right hand dye-injected neuron is a muscle spindle afferent (MSA), with a clear black ring. **B**: Cytoplasmic HCN2 relative intensities are elevated in MSAs versus other Aα/β neurons and all other neurons. **C**: HCN2 ring/edge staining is higher in MSAs than other Aα/β-neurons and than all other neurons; it is higher in Aβ-nociceptors than C-nociceptors, which showed no rings. Two out of three C-LTMs showed weak detectable ring staining. **D:** For non-MSA neurons ring intensities for HCN2 are highly significantly correlated with those for HCN1 (measured in different sections of the same neurons). For MSAs the linear regression was not significant (ns). The best linear regression line for MSAs was highly significantly elevated relative to non MSAs. **E**: HCN2 ring relative intensity is positively correlated with CV. For more detail and abbreviations see Fig. 2; for statistics see Methods.

Dye-injected neurons with identified sensory properties are shown in Fig. 3A before under fluorescence (upper row) and after (lower row) ABC HCN2-immunostaining. The C-unresponsive (nociceptor-type) neuron had no ring but patchy, weak cytoplasmic staining. The Aβ-nociceptor had no/weak ring and pale cytoplasm. The Aα/β-cutaneous LTM and the MSA both showed clear rings, but those of the MSAs were much denser; both had cytoplasmic staining. The three fine arrows show satellite cells devoid of HCN2 staining (as in Fig. 1A), confirming the ring staining as neuron membrane-related. Numbers of neurons are shown beneath columns in Fig. 3B; the 8 C-nociceptor neurons include 4 identified nociceptors (solid dots) and 4 nociceptor-type unresponsive neurons (star symbols). Objective scores for HCN2 ring staining were highly correlated with subjective scores (not shown), showing that quantitative scores are strongly related to directly observed ring intensity.

#### HCN2 cytoplasmic staining (Fig. 3B)

Most cytoplasmic values (both for individual neurons and medians of groups) fell below 20% of maximum ring intensity. The MSAs had the highest median cytoplasmic staining (39%), significantly (P<0.0001) higher than all other neurons, and than all other Aα/β-neurons. SAs also had a median just >20%, but there were only 3 neurons. Aα/β-neurons had higher medians than C neurons (P<0.01, not shown).

#### HCN2 ring immunointensity (Fig. 3C)

For all neurons, ring HCN2 staining intensities were positively linearly correlated with cytoplasmic intensities (P<0.0001, r^2^ = 0.72, plot not shown).

C-nociceptor-type neurons had no visible ring and very low HCN2 intensity at the cell edge. In contrast 2 of the 3 C-LTMs had weak but visible ring staining. The four Aδ-nociceptors had no detectable HCN2 ring staining (despite most having visible rings with HCN1 staining). 25 of 28 Aα/β-neurons had visible rings (20–100% intensity). Aβ-nociceptors had variable ring staining, significantly higher than C-nociceptor edge staining; the latter was similar to Aδ-nociceptor edge staining. In clear contrast to HCN1, HCN2 ring staining in MSAs was significantly greater than Aα/β cutaneous LTMs, and than all other Aα/β-neurons (P<0.001). In addition, it was significantly greater than in all other neurons (P<0.0001, not shown). Thus MSAs had both the highest ring and the highest cytoplasmic staining.

#### HCN2 versus HCN1-ring staining (Fig. 3D)

Immunocytochemistry for both HCN1 and HCN2 was carried out successfully on different sections of 37 dye-injected neurons. These were 4 C-nociceptors, 4 C-unidentified neurons, 3 Aδ-nociceptors, 0 Aδ-LTMs, 5 Aβ-nociceptors, 13 Aα/β-cutaneous LTMs and 8 MSAs. The ring intensities for both (each relative to the maximum ring intensity for that isoform) are plotted. They were highly correlated for the 29 non-MSA neurons. For MSAs there was no significant correlation. The best fit regression line for MSAs (dashed line) was significantly elevated compared to that for all non-MSAs. Thus for any given HCN1-ring intensity, the HCN2-ring intensity was greater in MSAs than in non-MSAs. That is, HCN2:HCN1 ratios were highest in MSAs.

HCN2 ring intensities were positively correlated with CV (Spearman’s correlation) for: all neurons; all A-neurons; all nociceptors (P<0.01, r_s_ = 0.62) and all LTMs including MSAs (P<0.05, r_s_ = 0.48) but not for C-neurons alone (Fig. 3E). Note that MSAs, which had the greatest HCN2 ring staining, also tended to have the fastest CVs.

### How do HCN1 and HCN2 Rings Relate to Published I_h_ in the Same Neuronal Groups?

**Figure 4:**
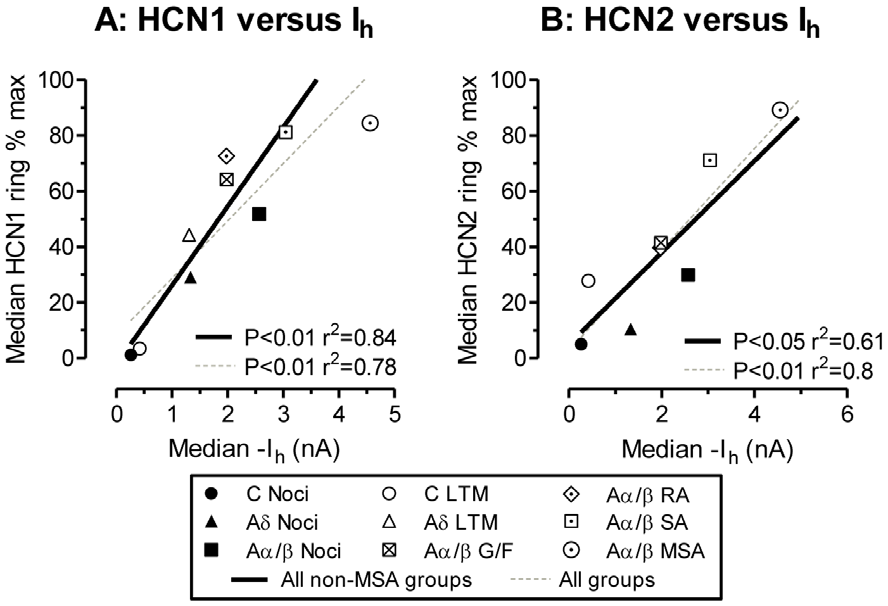
Ring staining for HCN1 and HCN2 versus I_h_ in different neuronal groups. Median values for HCN1 and HCN2 ring intensity for the different neuronal subgroups plotted against published median I_h_ values taken from Table 1 in Gao et al., 2012, for the same neuronal subgroups, thus each symbol represents medians from one subgroup. Note that unlike the immunocytochemistry, the I_h_ was measured in non-dye injected neurons. For ranges of values in each group see Fig. 2 and 3 and Gao et al 2012. Because I_h_ and HCN2 were both significantly higher in MSAs than for any other neurons, we show the linear regressions for all groups excluding MSAs (black lines) and for all groups including MSAs (grey dashed lines).

There are strong linear correlations between median HCN1 ring intensities (Fig. 4A) or HCN2 ring intensities (Fig. 4B) when plotted against (previously published) median I_h_ values (obtained from Table 1 in Gao et al., 2012 [10]) for the same neuron subtypes. Thus I_h_ magnitude is related to the HCN1 and HCN2 expression at the neuron perimeter. The slightly weaker correlations for HCN2 may result from fewer neurons including a lack of HCN2 data for Aδ LTMs. The Aδ-nociceptor median (filled triangle) falls close to the regression lines for HCN1, but falls below it for HCN2, suggesting that median I_h_ may be more closely related to membrane/ring HCN1 than HCN2 staining. The MSA point for HCN2 versus I_h_ falls close to the regression line for all non-MSA groups; including it in the analysis increased the r^2^ value. In contrast, the MSA HCN1 versus I_h_ point falls to the right of the non-MSA line; its inclusion in the correlation decreases r^2^ despite more data points. This suggests that median I_h_ in MSAs is more closely related to the median ring intensity for HCN2 than for HCN1.

### Effect of NT3 on HCN1 and HCN2 Expression *in vitro*

We observed that ring staining in medium-large DRG neurons of HCN1 and HCN2 immunoreactivity was much stronger *in vivo* than in cultures, both ours and published studies. I_h_ magnitudes are also greater *in vivo* than *in vitro* (see Introduction). This suggested that something was missing in the culture medium (which already included NGF) that may influence HCN1 and HCN2 expression. Because many large neurons express trkC and have NT3-dependent phenotypes [15], [16], [45]–[47], and because cultures used for studies of HCN1 or HCN2 or I_h_ are often maintained with NGF but without NT3, we hypothesised that HCN1 and HCN2 ring staining in medium/large neurons may be NT3-dependent. We therefore compared HCN1 and HCN2 immunostaining at the neuron edge (equivalent to the ring in DRG sections), in neurons in NT3+ (40 ng/ml NT3) versus NT3− (no NT3) cultures, all of which included NGF (10 ng/ml).

**Figure 5:**
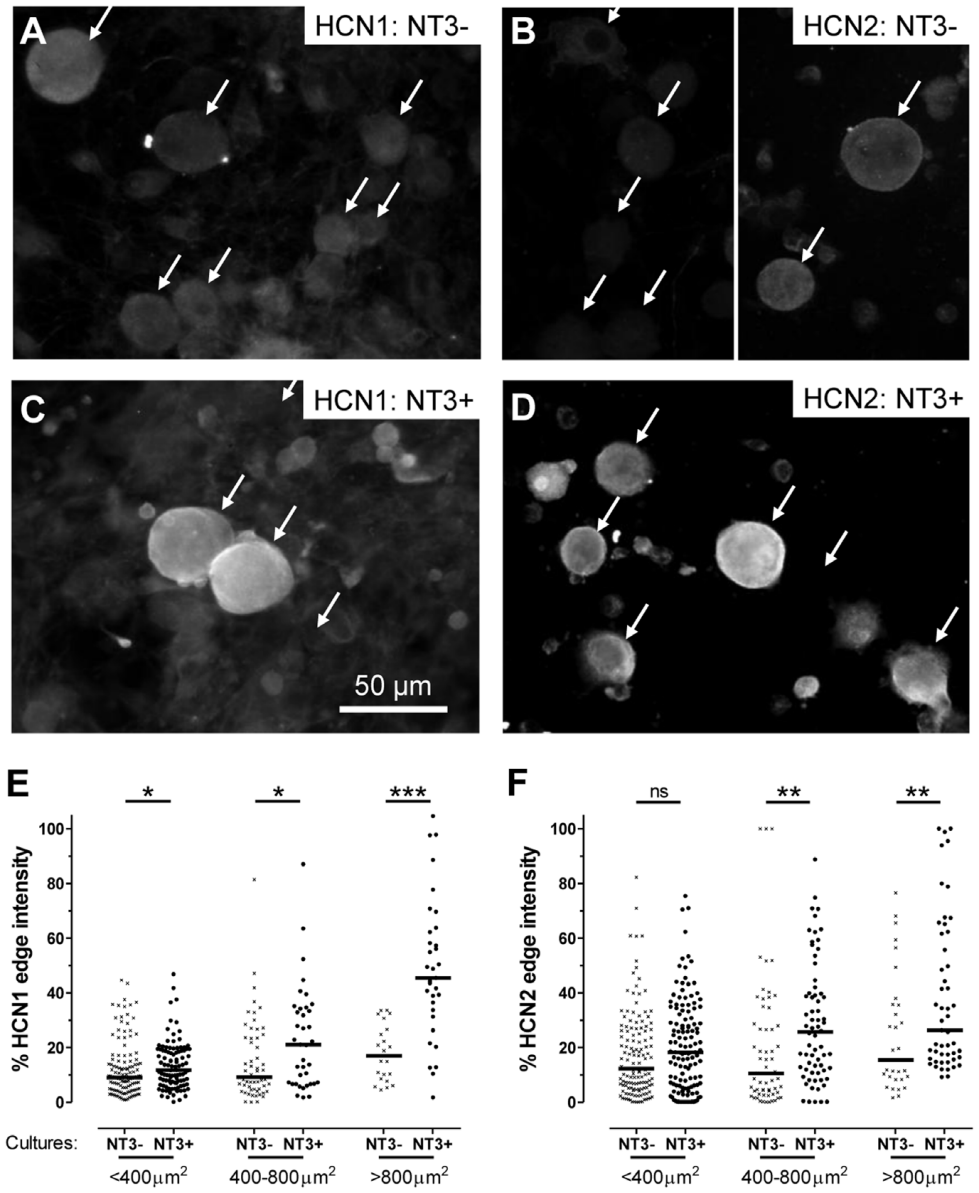
HCN1 and HCN2 dependence on NT3. **A to D**: DRG neurons cultured for 2 days in absence (**A** and **B**) or presence (**C** and **D**) of NT3 (40 ng/ml). HCN1 (A and C) and HCN2 (B and D) immunofluorescence examples are shown. Immunostaining for both was much reduced in the absence of NT3. Arrows in A and B show neurons visible under interference contrast; many were so weakly stained they were not/weakly visible under fluorescence. Note that in **B**, the left hand image includes smaller neurons; the right image includes a medium-sized and a large neuron. In **C** and **D** (with NT3), staining was stronger at the cell perimeter of medium to large neurons for both HCN1 and HCN2. Some smaller neurons showed elevated HCN2 staining (**D)**. **E** and **F**: Quantitation of the effect of NT3 (40 ng/ml) on HCN1 and HCN2 edge staining after 2 days in culture. Medians for HCN1 and HCN2-edge immunostaining were both significantly greater (Mann Whitney test) for medium and large neurons in the presence of NT3. For HCN1 only, there was also a significant elevation in edge staining in small neurons with NT3. * P<0.05; **P<0.01, ***P<0.001, ****P<0.0001.

Example images and image analysis are shown for 2 days cultures (Fig. 5). In the absence of NT3 (NT3−) immunostaining was very low for both HCN1 (Fig. 5A) and HCN2 (Fig. 5B) with no clear ring staining. Weak immunointensity in NT3− cultures meant that many neurons could only be seen under interference contrast, so arrows indicate healthy neurons seen under interference contrast.

Ring staining was less clear in whole cultured DRG neurons than in 7 µm DRG sections, due to their geometry. Nonetheless, neuronal rings (elevated perimeter staining) were evident for both HCN1 and HCN2 in NT3+ cultures. The ring/edge staining and staining throughout neurons in NT3+ cultures were dramatically increased both for HCN1 and HCN2 (Fig. 5C and D), on all days examined (1, 2, 3 and 4 days *in vitro*). The increased edge/ring immunostaining was significant for all sizes of neuron for HCN1, and very highly significant for large neurons (Fig. 5E). Increases for HCN2 were highly significant in medium and large neurons, but not in small neurons (Fig. 5F).

The ratio of large to small plus medium-sized neurons was greater with NT3 (84/339 for NT3+ cultures versus 52/386 for NT3− cultures, Fisher’s exact test, P = 0.0015) at 2 days in culture. Thus the large size of some neurons may be NT3-dependent. However, decreased NT3-dependent HCN1 and HCN2 expression was unlikely to depend on neuronal loss because a) there was little evidence of neuronal death up to 2 days; b) presence in NT3- cultures of large neurons with no/low staining (Fig. 5A and B) and c) reduction in NT3− cultures of immunostaining in neurons of all sizes (Fig. 5).

### Effects of Cutaneous Inflammation by CFA on HCN2 Expression

HCN2 has been implicated in inflammatory pain and more specifically in the excitability and spontaneous firing in C-fiber neurons following inflammation (see Introduction and Discussion). It was previously shown that HCN2 and I_h_ were both increased in small (C-nociceptive) neurons 5 and 7 days after CFA-induced cutaneous inflammation (CFA), and that at this time there was spontaneous firing that was I_h_-dependent [5]. However, because we previously showed that following CFA, spontaneous pain behaviour and spontaneous firing in C-nociceptors increased by day 1 but almost recovered by day 4 after CFA [17], we investigated whether HCN2 expression was altered in DRG neurons at these times after CFA.

**Figure 6:**
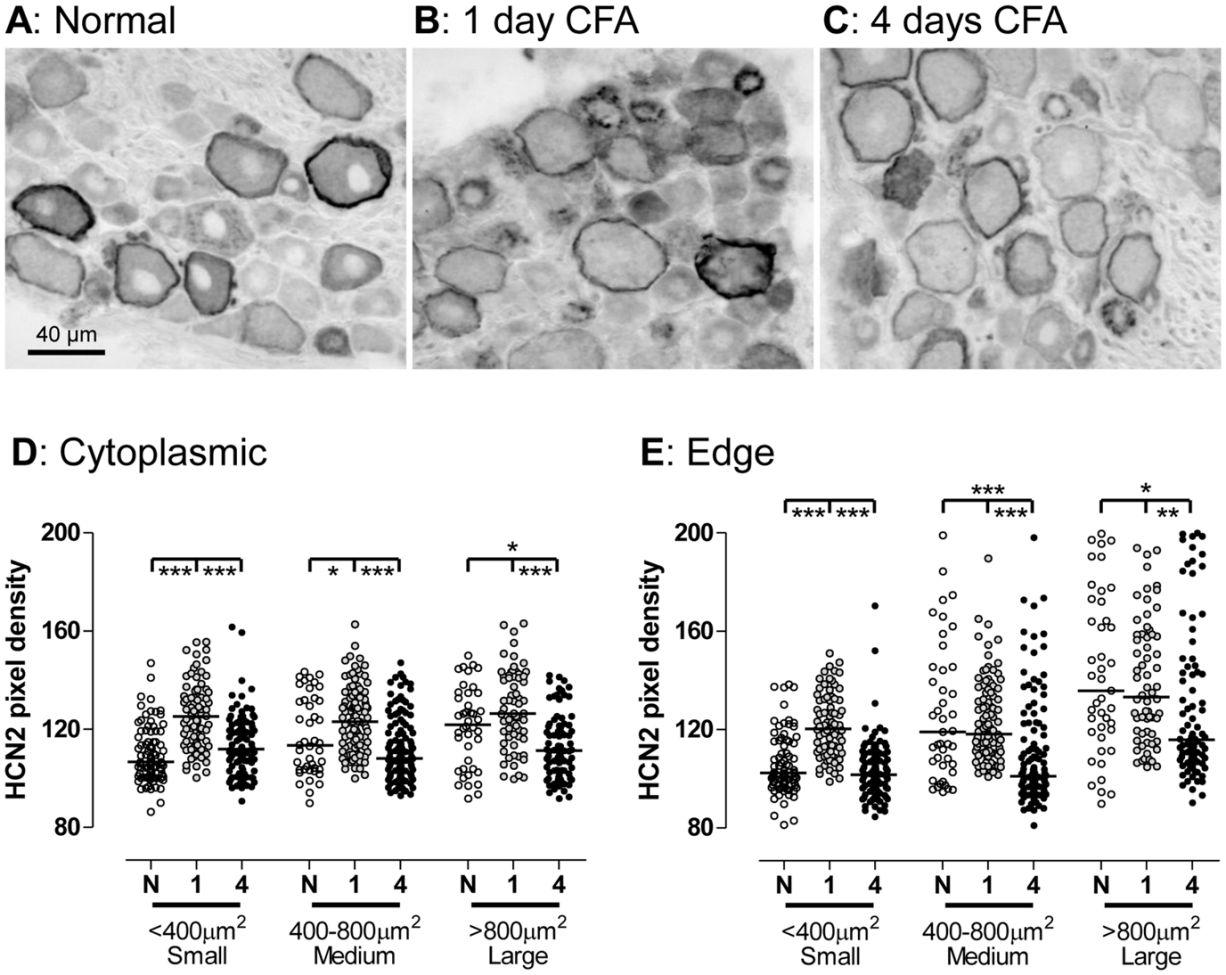
HCN2 immunointensity in L4 DRGs 1 and 4 days after inflammation. **A to C**: Representative images of ABC HCN2 staining in normal L4 DRG (**A**) and 1 day (**B**) or 4 days (**C**) after CFA-induced cutaneous inflammation. Note the elevation in the overall staining of small neurons by 1 day and the weaker HCN2 ring and cytoplasmic staining in medium and large neurons by 4 days. **D** and **E**: Neurons subdivided into small, medium and large (cross sectional areas with nuclear profiles of <400 µm^2^, 400–800 µm^2^, >800 µm^2^ respectively). Cytoplasmic staining: pixel densities over entire cytoplasm; edge staining: pixel densities under line drawn over cell perimeter. Comparison of cytoplasmic (**D**) and edge (**E**) staining in sections of normal (N) L5 DRGs with ipsilateral L5 DRGs 1 day (1) and 4 days (4) after CFA-induced cutaneous inflammation. Kruskall-Wallis of N, 1 and 4 groups within each size range with Dunn’s post-hoc test between each possible pair. For significances see Fig. 5 legend.

There were clear differences in the appearance of HCN2 immunostaining 1 and 4 days after CFA compared with untreated rats (Fig. 6A to C). The obvious changes include increased intensity in small and medium sized neurons with clear cytoplasmic patches/blobs (but no rings in small neurons) at 1 day recovering by 4 days, and decreased ring and cytoplasmic staining in large neurons by 4 days but no change by day 1. These changes were significant or highly significant compared with untreated rats (Fig. 6D and E). In small neurons, the edge/ring staining changed exactly as for cytoplasmic staining, increasing by 1 day and recovering to normal by 4 days (Fig. 6B). This similarity between ring and cytoplasmic staining accords with the lack of visible rings in these neurons.

### Effects of Cutaneous Inflammation by CFA on NT3 Expression in DRGs

By 1 day after cutaneous infammation induced by CFA, NGF has increased in skin and DRG [48], [49]. This may (see Discussion) be a cause of the increased HCN2 in small DRG neurons 1 day after CFA. The decreased HCN2 expression in large neurons 4 days after CFA-induced cutaneous inflammation may result from decreased NT3 levels because a) NT3 decreases in skin by 2 days after CFA [49] and b) we have demonstrated NT3-dependence of HCN2 expression especially in large neurons. However, NT3 levels in the DRG after cutaneous CFA-induced inflammation have not previously been examined. We therefore examined next whether NT3 also decreases in the DRGs.

#### Western blots

**Figure 7:**
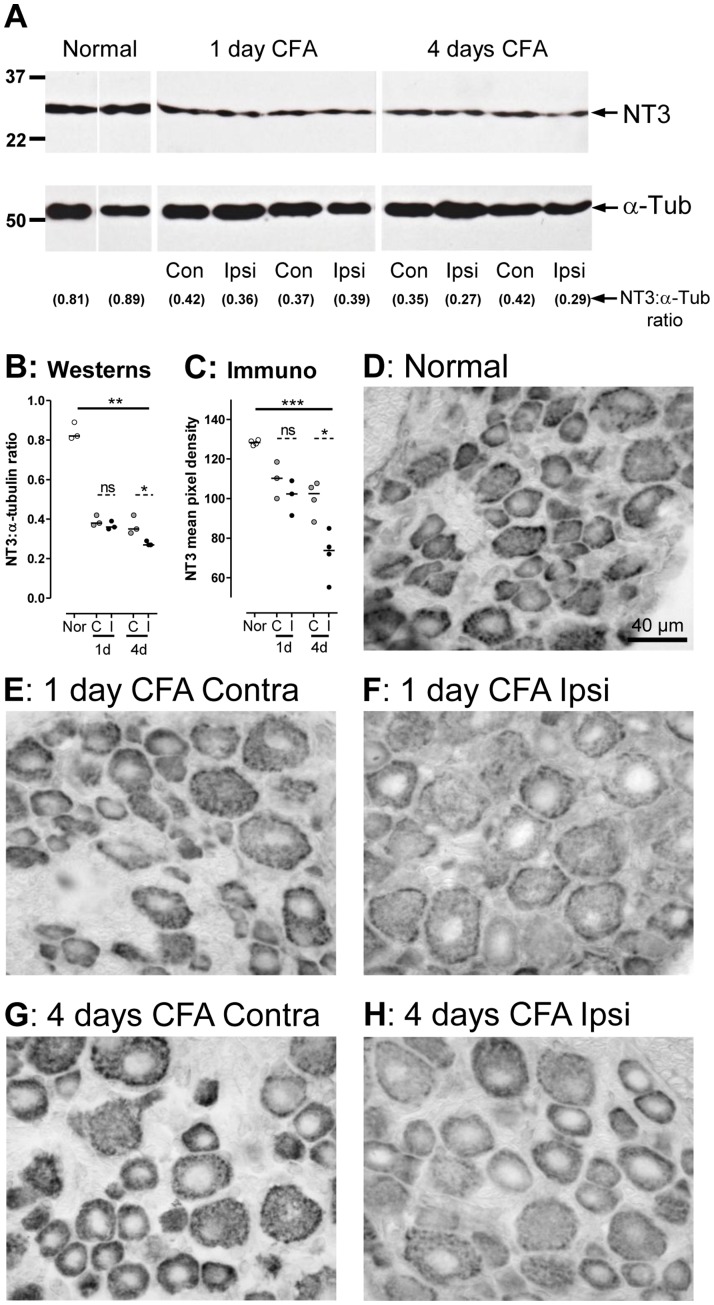
NT3 expression in DRGs 1 and 4 days after Inflammation. **A**: Western blots of pools of 2 DRGs (L4 and L5) from one rat per pool. Upper: NT3 examples from two normal rats, two 1 day CFA rats (ipsilateral DRGs) and two 4 day CFA rats. Lower: the α-tubulin Westerns on the same lane as the NT3 as loading controls. The NT3 has decreased at both 1 and 4 days, both ipsilaterally and contralaterally. Below, the ratios of pixel densities (NT3:α-tubulin). **B:** Plots of these ratios confirm this (one symbol per rat) and show a significant decrease after 4 days in NT3 ipsilaterally (I) relative to contralaterally (C) and to normal (Nor). **C:** Graph of NT3 staining: Each symbol shows the average pixel densities for one rat from 9 regions from capured images (examples in **D–H**) of a mid-section of an L4 DRG. The plot confirms the findings in **B**. Nor, C and I defined as for **B**. It shows a highly significant reduction 4 days after cutaneous CFA-induced inflammation ipsilaterally compared with both contralaterally and with normal. Non-significant decreases in immunoreactivity after 1 day ipsilaterally and contralaterally and after 4 days contralaterally can also be seen. **D** to **H:** ABC immunostaining for NT3 in L4 DRGs was most intense in normal rats and least intense ipsilaterally 4 days after CFA-induced inflammation. The staining was in all cell sizes, and diminished in all cell sizes after inflammation.

NT3 levels were compared in DRGs form normal and inflamed rats 1 and 4 days after CFA. L4 and L5 DRGs were taken and pooled from each of three rats in each of the three groups: untreated (normal), and from ipsilateral and contralateral sides of the inflamed rats. Ipsilateral and contralateral pools were kept separate, as were pools from different rats. In all rats, NT3 bands were clearly diminished compared to normal both 1 and 4 days after intradermal CFA injection (Fig. 7A), in both ipsilateral and contralateral DRGs. A plot of the ratio of NT3:α-tubulin gives a semi-quantitative indication of the changes (Fig. 7B) with a decrease ipsilaterally after 4 days, significantly different from normal and from the contralateral values.

#### Immunostaining

Median pixel densities per rat for 9 regions in the L4 DRGs show the following. The NT3 immunostaining intensity was strong normally, apparently decreased somewhat by 1 day post CFA, but this was not significant (only 3 rats); it decreased highly significantly by 4 days after CFA in the ipsilateral L4 DRGs (Fig. 7C). These changes are apparent in the photomicrographs (Fig. 7D to H).

## Discussion

This is the first report of the expression of HCN1 and HCN2 in DRG neurons with identified sensory properties *in vivo*. The expression of these isoforms in different neuronal subtypes can explain both the previously reported I_h_ magnitudes and I_h_ properties [10] in these subgroups. Expression of both isoforms was NT3-dependent. CFA-induced skin inflammation increased HCN2 expression in small neurons by 1 day but decreased it in large neurons by 4 days.

This is also the first quantification of HCN1 and HCN2 ring staining. The absence of staining over satellite cells showed this ring to be membrane-associated, as it is in trigeminal neurons [40]. Factors contributing to ring intensity probably include density of channels in the soma membrane, extent of membrane folding [50] and/or channels within vesicles near the membrane.

A striking difference between different neuronal subgroups is the ring staining present in A-neurons, but absent in C-nociceptors. The concentration of HCN1 and HCN2 at nodes of Ranvier may indicate mechanism/s that concentrates these proteins in naked nodal (non-myelin-covered) membrane regions of A-neurons. Similar mechanism/s could send both isoforms towards the soma membrane in A-neurons. In those C-LTMs with visible HCN2 rings, HCN2 may also be sorted to the soma membrane with similar mechanism/s. The lack of rings in C-nociceptors suggests they lack such mechanism/s.

### HCN1, HCN2 and I_h_ in Different Sensory Groups

The strong linear correlations between median I_h_ and both HCN1 and HCN2 ring intensities, plus the similarities between extent of scatter of values for I_h_ [10], HCN1 and HCN2 ring intensities, are entirely consistent with these isoforms underlying the majority of I_h_ in these different neuronal subgroups. Differences between levels of HCN1 and HCN2 isoform expression can, however, be seen in some neuronal subgroups.

The greater I_h_ in MSAs [10] appears related to, and may depend on, their elevated HCN2 expression shown here. This high HCN2 in MSAs could explain previous findings [9] that neurons with diameter >50 µm had more abundant mRNAs for HCN2 than HCN1, since such large neurons include a high proportion of MSAs.

The elevated I_h_ in some C-LTMs [10] may result from the elevated HCN2 edge staining in some of these neurons; however with only one C-LTM we cannot comment on HCN1. In Aδ-nociceptors, HCN1 ring relative staining is a) higher than for HCN2 and b) apparently better related to median I_h_ than HCN2 suggesting that I_h_ may be more closely related to membrane/ring HCN1. This accords with the fast I_h_ activation time constant in Aδ-nociceptors consistent with that of HCN1 [10].

The overall similarities and discrete differences (above) in the HCN1 and HCN2 distributions, can thus account for the distributions of I_h_ *in vivo* [10] in the different neuronal subgroups. Our data strongly suggest that ring intensity is indicative of the amount of functional channel.

### HCN Isoforms in Relation to I_h_ Properties

The reported properties of I_h_ in different neuronal subgroups reported by Gao et al., 2012 [10] are also in accordance with HCN1 and HCN2 expressions. In that study, both activation time constants and half activation voltages (V_0.5_) for I_h_ indicated contributions from both HCN1 and HCN2, probably mainly in the form of HCN1+HCN2 heteromers in the presence of cAMP. This suggests that the co-expression of HCN1 with HCN2 may result in formation of co-assembled HCN1+HCN2 heteromers, as occurs in hippocampal neurons [51], [52]. The loss in HCN2−/− mice of the cAMP sensitivity of I_h_ V_0.5_ [3], in small, medium and large cultured DRG neurons confirms a contribution of HCN2 to I_h_ in DRG neurons normally in all CV groups.

### Potential Functional Implications of HCN1 and HCN2 Co-expression

Properties of I_h_ associated with heterologously expressed HCN1 or HCN2, show the following (reviewed in [1]). HCN1-related I_h_ has a) a relatively depolarised V_0.5_ (∼−66 mV) and b) the fastest activation of all HCN homomeric channels. Furthermore, surface/membrane expression of HCN1, not HCN2, increases within minutes in response to neural activity (caused by glutamate applied to hippocampal neurons [53]). In contrast to HCN1, HCN2-related I_h_ in the absence of cAMP has a) a more hyperpolarised V_0.5_ (∼−92 mV) and b) a slower activation than for HCN1. HCN2-related I_h_ (but not HCN1-related I_h_) is highly modulated by cAMP which: a) increases I_h_ magnitude by increasing HCN2 open probability [54], [55]; b) depolarises V_0.5_ (by ∼15 mV) and c) accelerates I_h_ activation [1]. I_h_ due to HCN1+HCN2 heteromeric channels has properties intermediate between those of HCN1 and HCN2-related I_h_. HCN1+HCN2 channels have activation kinetics that are closer to HCN1, a voltage dependence (V_0.5_) closer to HCN2 and an intermediate dependence on cAMP. HCN1+HCN2 heteromers have a much greater single channel conductance (9 pS) than HCN2 alone (1.5 pS) and somewhat greater than HCN1 alone (variable from <1 to 8 pS in different cell types) [54], [56], [57].

In DRG neurons, any I_h_ due to HCN1 homomers, would normally be partly activated contributing a small I_h_ at DRG neuron resting Em (typically −50 to −60 mV). The mean afterhypolarization (AHP) depth in DRG neurons recorded *in vivo* with KCl-filled electrodes is −65 mV±8.7 (SD, n = 342), with 25^th^ and 75^th^ percentiles of −60 mV and −71 mV respectively (unpublished observations, this group). Thus at mean AHP depth the HCN1-related I_h_ would be ∼50% activated, generating a larger I_h_ during the AHP, probably accelerating Em recovery and thus facilitating repetitive firing. However if activity results in substantial HCN1 recruitment to the membrane the above effects would be greatly enhanced during periods of activity. Similarly, any HCN2 homomers are unlikely to contribute to I_h_ at resting Em and only to contribute slightly during the AHP. Increased cAMP resulting from activity and/or inflammation would result in a greater contribution especially during the AHP, but even so, this current may be small due to the low HCN2 homomer channel conductance.

Our previous evidence [10] showed that the I_h_ in most DRG neurons has properties indicative of functional HCN1+HCN2 heteromers (see above), but did not preclude contributions to I_h_ of homomeric HCN1 and/or HCN2 channels. This would add DRG neurons to the list of CNS neurons for which there is evidence for functional HCN1+HCN2 heteromers [52], [58]. Importantly, the greater unitary conductance of HCN1+HCN2 heteromers could make this the predominant I_h_, especially in the presence of elevated cAMP and/or during neural activity.

MSAs have very high HCN2 and high HCN1 expression, and most of those recorded here had ongoing activity. This activity may well recruit more HCN1 into the membrane, perhaps enabling greater HCN1+HCN2 heteromerisation; it would also elevate cAMP which would increase HCN2-related and/or HCN1+HCN2-related I_h_. Together the high HCN2 and these activity-related changes may account for the much larger I_h_ measured in most MSAs [10].

From the above, it seems that changes in I_h_ during activity and/or inflammation may be very important for dynamic modulation of a variety of sensory neuron functions.

### NT3-dependence of HCN1 and HCN2 Expression

The NT3-dependence of immunostaining for both HCN1 and HCN2 in DRG neurons could explain the low published I_h_ values (Introduction) in medium/large DRG neurons in cultures lacking NT3. Interestingly, the cytoplasmic HCN1 staining in small neurons was also NT3-dependent. Most small neurons do not normally express trkC, the high affinity NT3 receptor [16]. However, NT3 also binds to trkA, which is expressed on many small C-fiber DRG neurons [59], [60]. Thus in small neurons the effect of NT3 on HCN1 (but not HCN2) expression could be trkA-mediated (although NGF may also affect HCN1 expression via trkA). The NT3 dependence of HCN1 expression in all sizes of DRG neuron and of HCN2 in medium to large neurons highlights the importance of adding NT3 to cultures of DRG neurons used for studies of HCN channels and I_h_. This NT3-dependence is the first evidence for modulation of HCN1 and HCN2 expression in DRG neurons by trophic factors.

### Effects of Altered HCN2 Expression during Inflammation on Neurons and Inflammatory Pain

The following evidence supports HCN2 being important in inflammatory pain [3], [5], [61]. Comparison of small DRG neurons in cultures from wildtype or HCN2−/− mice, indicates that the increased firing in the presence of PGE2 or forskolin (which both cause elevated cAMP) is due to HCN2 [3]. In addition, 5–7 days after intracutaneous CFA, C-nociceptors showed elevated I_h_, increased HCN2 expression, and spontaneous firing that was reduced by the I_h_ blocker ZD7288 [5], were both reported, suggesting an important role for HCN2 5–7 days post CFA. However, the greatest CFA-induced spontaneous firing in C-nociceptors is 1day after CFA, declining by 4 days [17]. The much greater HCN2 upregulation that we report in small (mostly nociceptive) DRG neurons at 1 day than at 4 days after CFA is thus consistent with HCN2 contributing to their much greater spontaneous firing at 1 day. This HCN2 upregulation may also enhance the acute effects of inflammation-induced elevation of cAMP levels eg [62], [63], further increasing the firing.

Effects of decreased HCN2 by 4 days post CFA on large DRG neurons and their fibers are, however, harder to predict. Large neurons include Aβ-nociceptors and Aα/β-LTMs, but our CFA study (Fig. 6) did not distinguish between these. It is likely that cutaneous inflammation would affect cutaneous afferents. Muscle LTMs are thus much less likely to be affected. Note that a subgroup of large neurons retains high HCN2 even 4 days after CFA; these may well be the highly HCN2-expressing MSAs.

### Possible Causes of Altered HCN2 Expression in DRG Neurons after CFA

The above HCN2 changes and their timescales after CFA indicate different controls of HCN2 expression between small and large neurons. These could potentially be explained by trophic factor expression patterns such as NGF and NT3 in skin as these are both retrogradely transported to DRG neurons [64]. After intradermal CFA injections, the expression levels in the skin change for both. NGF increases in skin, peaking at 6 hrs, and thereafter declining towards normal [49]; NGF levels also increase (doubled) in the DRG after 1 day then decline towards normal from day 2 to day 7 [48]. NT3 after CFA, in contrast to NGF, declined in CFA-inflamed skin to ∼50% of normal levels by 12hrs and continued to decline to at least 2 days [49]; we show here that NT3 levels also decreased in the DRG 4 days after CFA.

The increased HCN2 in small DRG neurons at 1day is unlikely to result from altered NT3 levels, because a) the NT3 decreases but HCN2 increases; b) the NT3 change is greater at 4 days when HCN2 levels are almost recovered; c) most small neurons do not express trkC, the high affinity receptor for NT3 receptor [16] and d) because HCN2 expression in cultured small neurons is not NT3-dependent (this paper). However, since many HCN2-expressing C-fiber neurons express trkA (Fig. S3), and NGF increases with a similar time course (see above), NGF may act via trkA to increase HCN2 in small neurons 1 day after CFA. This needs to be tested.

In contrast, medium and large neurons normally have NT3-dependent (this paper) HCN2 expression. At 4 days after CFA, their decreased HCN2 is thus consistent with decreased NT3 in skin and DRG. Although these trophic factors seem likely to contribute to HCN2 changes in small and/or large neurons after CFA, other contributory factors cannot be excluded.

## Conclusions

HCN1 and HCN2 are expressed in a variety of sensory neuron types; the expression is highly related to CV, being highest in Aα/β neurons both LTMs and nociceptors. Their expression levels are consistent with them underlying both I_h_ magnitude and I_h_ properties in all neuronal subgroups examined. Expressions of both HCN1 and HCN2 are dependent on NT3. These patterns of HCN1 and HCN2 immunostaining plus our previously reported I_h_ properties in identified neurons *in vivo*, coupled with the NT3-dependence of their expression, can explain many of the previously published observations of I_h_, including those obtained *in vitro*.

The very high I_h_ in MSAs may relate to their particularly high HCN2 and their high levels of activity. Inflammation-induced upregulation of HCN2 in small putative C-nociceptors could well contribute to chronic inflammatory pain.

Finally, because of the probable contribution of HCN2 to I_h_ in a variety of sensory neuronal subtypes, modulation of HCN2 function/expression in DRG neurons (for example to prevent or treat inflammatory pain) could potentially influence several sensory functions, including e.g. touch and proprioception (MSAs). Further studies on the *in vivo* functions and expression control mechanisms of HCN2 and HCN1 normally and during inflammation in different DRG neuronal subtypes are therefore needed.

## Supporting Information

Figure S1 **Characterization of HCN1 and HCN2 antibodies. A and B:** Western blots of HCN1 and HCN2 in whole brain, liver, kidney, spinal cord and DRG. A: HCN1 antibody shows strong bands at expected molecular weight; B: HCN2 antibody shows ∼5 bands in brain tissue, but one strong band at the correct molecular weight in DRG tissue (arrows, ∼110 kDa). The bands at 55 kDa for both HCN2 and HCN1 may be breakdown products. **C–F:** Antibody preabsorption with HCN1 (**C, E**) and HCN2 (**D, F**) blocking peptides. Arrows indicate staining on adjacent sections of the same cells. Images are bright field photomicrographs with X40 objective, condenser position adjusted to show neuronal edges even where ring staining was absent. For both antibodies there was loss of ring and cytoplasmic staining with appropriate blocking peptides. C and D were stained with anti-HCN1 antibody; E and F were stained with anti-HCN2 antibody. C and D are adjacent sections, as are E and F. The unaltered staining with blocking peptide to the wrong antibody shows that HCN1 and HCN2 antibodies clearly distinguish between these two isoforms.
(TIF)

Figure S2 **HCN2 Antibody characterisation by siRNA knockdown. A**: HCN2 siRNA caused a reduction in mRNA level of HCN2 but not HCN1 or GAPDH in 1 day cultures treated with HCN2 siRNA, compared to scrambled siRNA (scr). **B**: Correspondingly, in DRG neurons cultured with NT3, HCN2 siRNA reduced HCN2 staining (right), compared with scr (left). Note that with HCN2 siRNA, the strongest staining was in (non-transfected) FAM negative neurons (open arrows), not in (transfected) FAM positive neurons (solid arrows). Photomicrographs (x20 objective). **C**: 1 day cultures, only transfected neurons were measured (>20% FAM cytoplasmic staining). HCN2 edge staining intensity (log scale) was highly significantly decreased (Mann-Whitney test, P<0.001) with HCN2 siRNA compared with scr treatment. These studies show selectivity of the HCN2 antibody for the HCN2 perimeter (edge) staining in DRG neurons.
(TIF)

Figure S3 **Double fluorescence immunostaining in L5 DRGs.** Interference contrast images (left) show neuronal outlines. HCN1 and HCN2 immunostaining in medium to large diameter neurons shows a clear ring over the neuronal perimeter. Symbols indicate examples of staining with: ‡ both antibodies, x neither, o clear HCN1 or HCN2 ring but not neurofilament or IB4. **A** (HCN1) and **B** (HCN2) with antibody RT97 to neurofilament (NF) shows clear ring staining only in NF-rich neurones; a few NF-poor neurons show cytoplasmic staining with the HCN2 antibody. **C** (HCN1) and **D** (HCN2) staining with IB4 conjugated to Alexa 488 (Invitrogen, UK) show that ring staining for both is in IB4-ve neurons, and HCN2 cytoplasmic staining is evident in two small neurons with strong and weak IB4 staining (see higher magnification in insets, wide = 45 µm×height = 36 µm). All images captured at 40X magnification. Note that of the small neurons with C-fibre (NF poor) with clear cytoplasmic HCN2 staining, ∼21% were IB4+ve. The remaining IB4-ve C-fibre neurons are likely to be trkA+ve [26].
(TIF)

Methods S1 (DOC)
