# Supplementary figures and images for: HCN1 and HCN2 in Rat DRG Neurons: Levels in Nociceptors and Non-Nociceptors, NT3-Dependence and Influence of CFA-Induced Skin Inflammation on HCN2 and NT3 Expression
Source: PLoS One. 2012 Dec 7;7(12):e50442. doi: 10.1371/journal.pone.0050442 (PMC3517619; doi:10.1371/journal.pone.0050442)

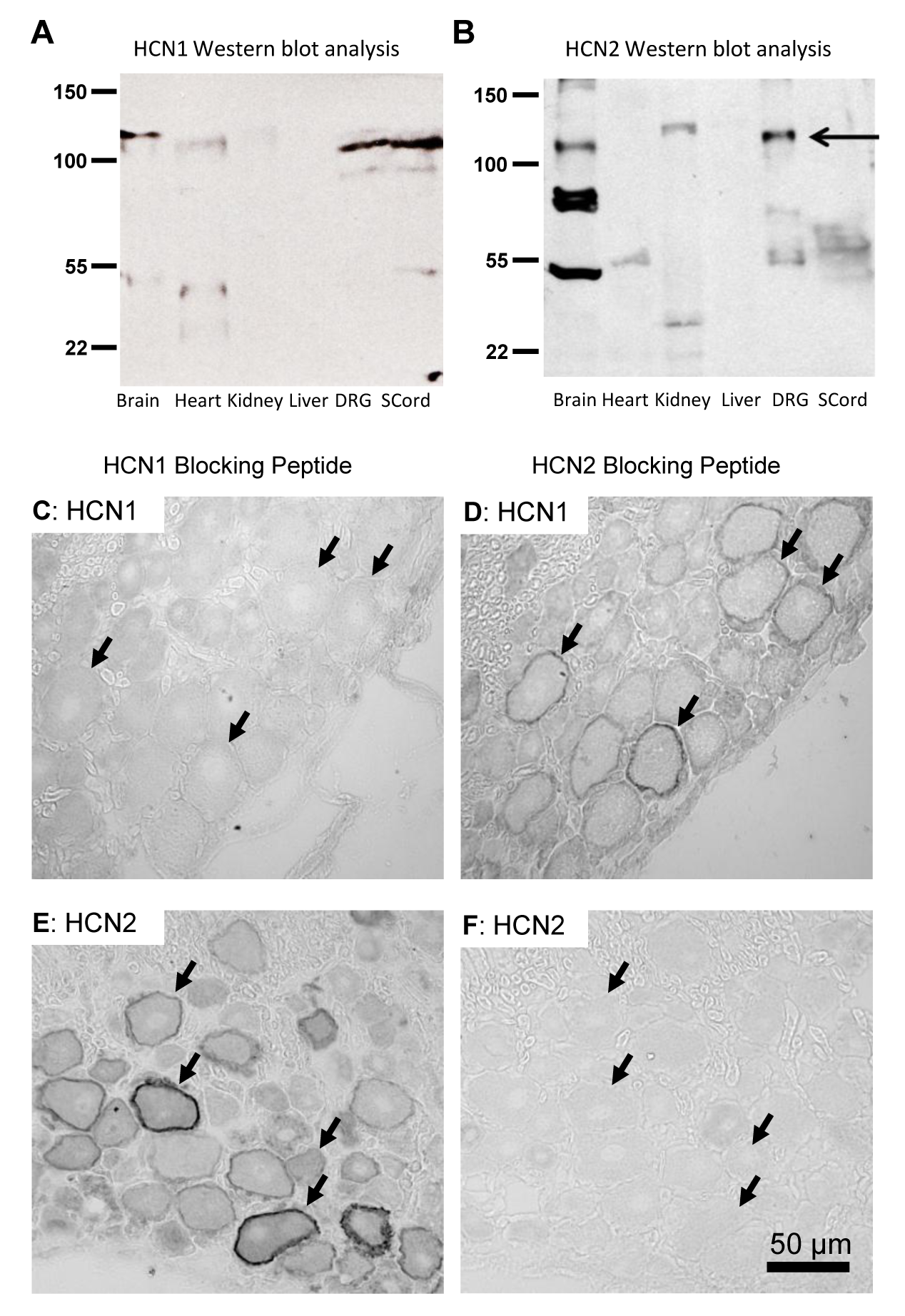

Supplement: Figure S1 — Characterization of HCN1 and HCN2 antibodies. A and B: Western blots of HCN1 and HCN2 in whole brain, liver, kidney, spinal cord and DRG. A: HCN1 antibody shows strong bands at expected molecular weight; B: HCN2 antibody shows ∼5 bands in brain tissue, but one strong band at the correct molecular weight in DRG tissue (arrows, ∼110 kDa). The bands at 55 kDa for both HCN2 and HCN1 may be breakdown products. C–F: Antibody preabsorption with HCN1 (C, E) and HCN2 (D, F) blocking peptides. Arrows indicate staining on adjacent sections of the same cells. Images are bright field photomicrographs with X40 objective, condenser position adjusted to show neuronal edges even where ring staining was absent. For both antibodies there was loss of ring and cytoplasmic staining with appropriate blocking peptides. C and D were stained with anti-HCN1 antibody; E and F were stained with anti-HCN2 antibody. C and D are adjacent sections, as are E and F. The unaltered staining with blocking peptide to the wrong antibody shows that HCN1 and HCN2 antibodies clearly distinguish between these two isoforms. (TIF) [file pone.0050442.s001.tif]

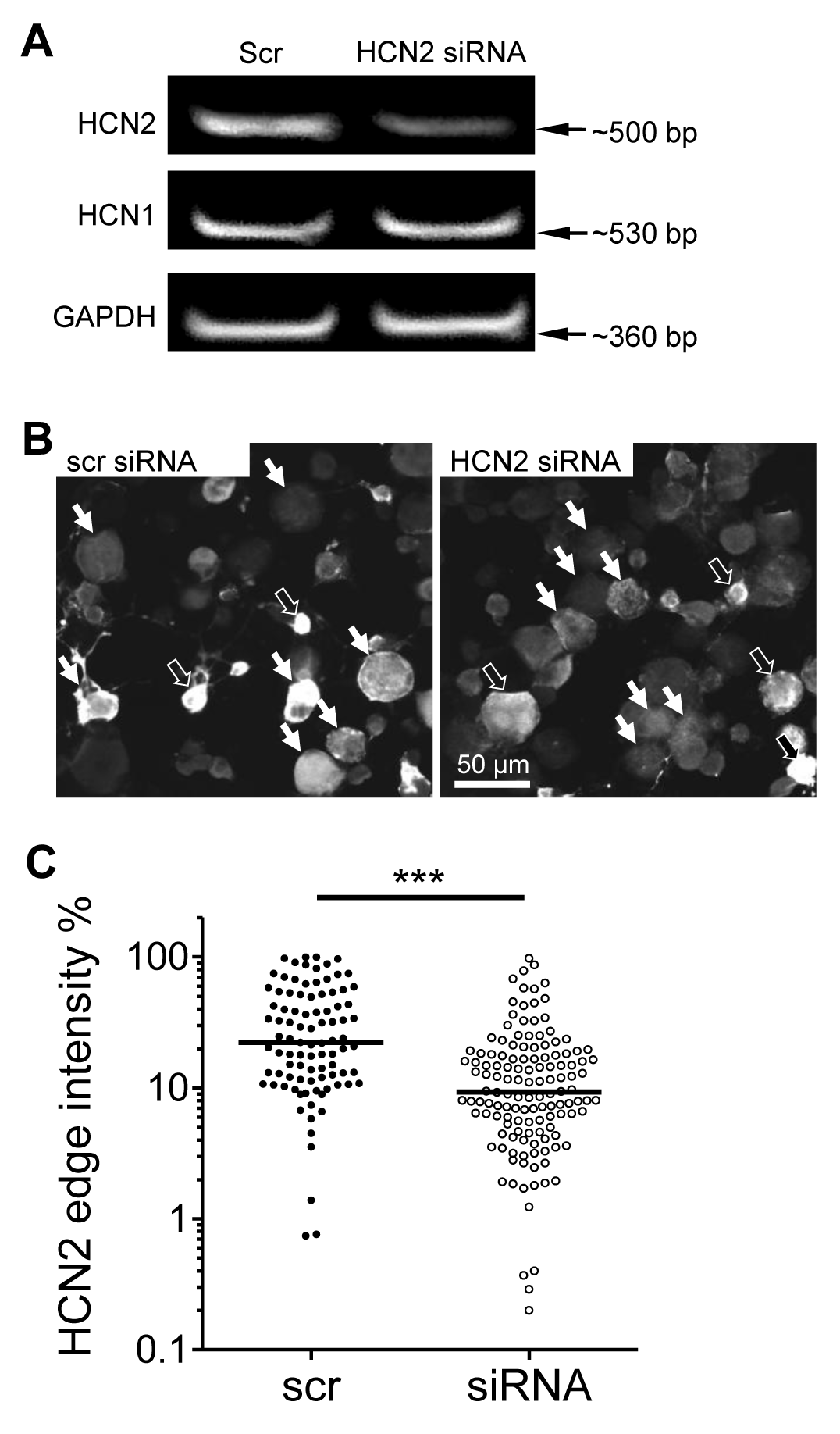

Supplement: Figure S2 — HCN2 Antibody characterisation by siRNA knockdown. A: HCN2 siRNA caused a reduction in mRNA level of HCN2 but not HCN1 or GAPDH in 1 day cultures treated with HCN2 siRNA, compared to scrambled siRNA (scr). B: Correspondingly, in DRG neurons cultured with NT3, HCN2 siRNA reduced HCN2 staining (right), compared with scr (left). Note that with HCN2 siRNA, the strongest staining was in (non-transfected) FAM negative neurons (open arrows), not in (transfected) FAM positive neurons (solid arrows). Photomicrographs (x20 objective). C: 1 day cultures, only transfected neurons were measured (>20% FAM cytoplasmic staining). HCN2 edge staining intensity (log scale) was highly significantly decreased (Mann-Whitney test, P<0.001) with HCN2 siRNA compared with scr treatment. These studies show selectivity of the HCN2 antibody for the HCN2 perimeter (edge) staining in DRG neurons. (TIF) [file pone.0050442.s002.tif]

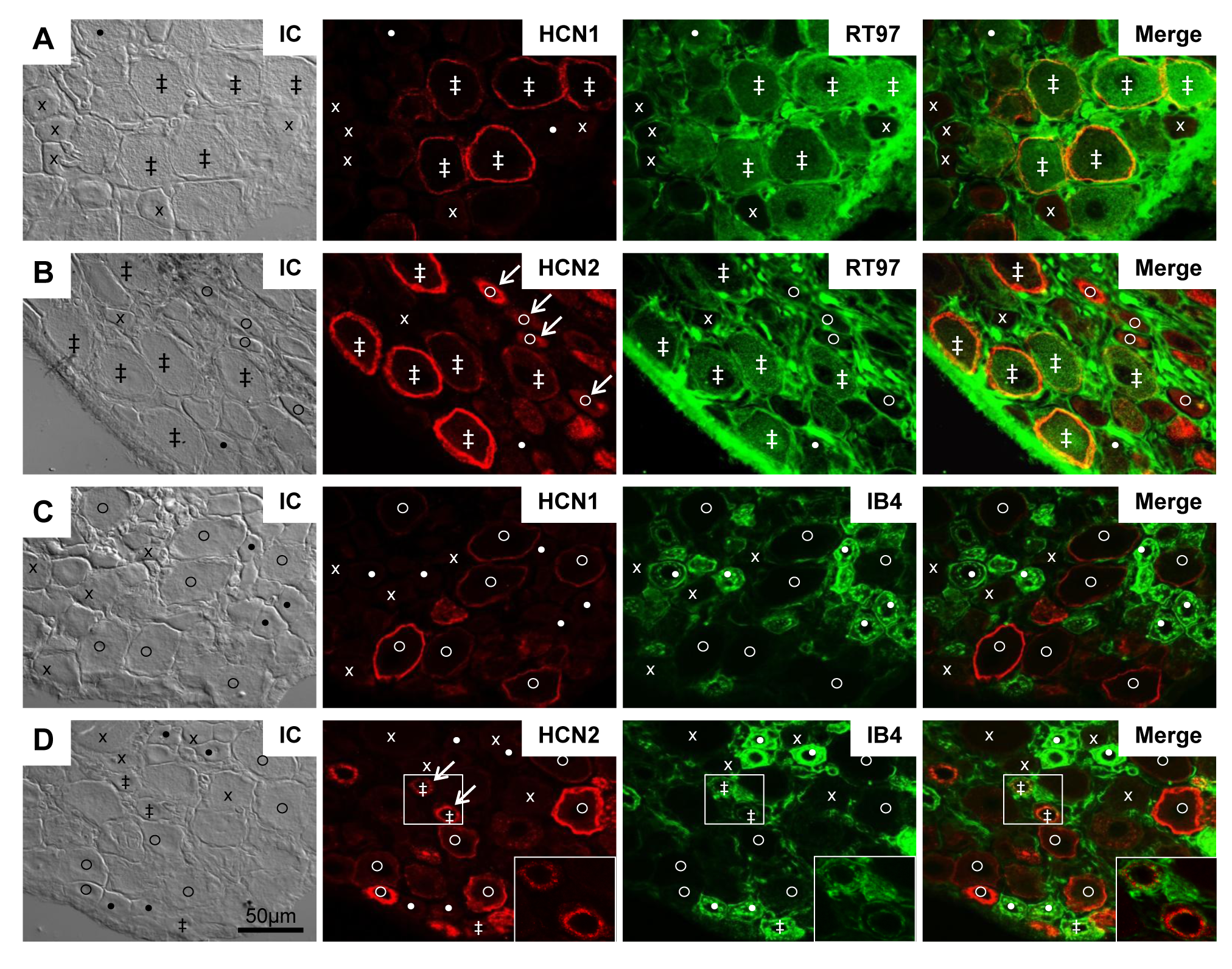

Supplement: Figure S3 — Double fluorescence immunostaining in L5 DRGs. Interference contrast images (left) show neuronal outlines. HCN1 and HCN2 immunostaining in medium to large diameter neurons shows a clear ring over the neuronal perimeter. Symbols indicate examples of staining with: ‡ both antibodies, x neither, o clear HCN1 or HCN2 ring but not neurofilament or IB4. A (HCN1) and B (HCN2) with antibody RT97 to neurofilament (NF) shows clear ring staining only in NF-rich neurones; a few NF-poor neurons show cytoplasmic staining with the HCN2 antibody. C (HCN1) and D (HCN2) staining with IB4 conjugated to Alexa 488 (Invitrogen, UK) show that ring staining for both is in IB4-ve neurons, and HCN2 cytoplasmic staining is evident in two small neurons with strong and weak IB4 staining (see higher magnification in insets, wide = 45 µm×height = 36 µm). All images captured at 40X magnification. Note that of the small neurons with C-fibre (NF poor) with clear cytoplasmic HCN2 staining, ∼21% were IB4+ve. The remaining IB4-ve C-fibre neurons are likely to be trkA+ve [26]. (TIF) [file pone.0050442.s003.tif]
