# Supplementary material for: HCN1 and HCN2 in Rat DRG Neurons: Levels in Nociceptors and Non-Nociceptors, NT3-Dependence and Influence of CFA-Induced Skin Inflammation on HCN2 and NT3 Expression
Source: PLoS One. 2012 Dec 7;7(12):e50442. doi: 10.1371/journal.pone.0050442 (PMC3517619; doi:10.1371/journal.pone.0050442)
Supplement: Methods S1 — (DOC) [file pone.0050442.s004.doc]

**METHODS S1**

**Characterization of HCN1 and HCN2 antibodies**

***Western blot****:* Whole protein extracts were obtained using Laemmli buffer and quantified by the Bradford method. Approximately 20 µg of protein were loaded into each lane**.** Expression of α-tubulin was used as a loading control. Anti-HCN1 (1:500), anti-HCN2 (1:1000) from Alomone labs, anti-NT3 (1:1000) from Abcam and monoclonal anti-α -tubulin (1:2000) from Sigma were used. Proteins were run in 8-12% PAGE gels and were transferred at 4ºC onto PVDF membranes (Immobilon-P, Amersham Biosciences, UK). Western blots were then developed using a chemoluminiscence kit (ECL plus, Amersham Biosciences, UK).

***Antibody preabsorption:*** primary antibodies anti-HCN1 (1:1000) and anti-HCN2 (1:2000) were preincubated in PBS for 1h at room temperature with specific blocking peptides for both antibodies (1:100, Alomone labs). Then adjacent mid-L5 DRG sections were processed for ABC immunocytochemistry as described in the main Methods section. Sections were therefore incubated with either anti-HCN1 or anti-HCN2 while their corresponding adjacent sections were incubated with the pre-absorbed antibodies. Thus there were 4 conditions to be examined: anti-HCN1 antibody preabsorbed against HCN1; anti-HCN1 antibody preabsorbed against HCN2; anti-HCN2 antibody preabsorbed against HCN1 and anti-HCN2 antibody preabsorbed against HCN2.

**HCN2 Antibody characterisation by siRNA knockdown**

***siRNA knockdown***: Pre-designed Silencer® Select siRNA against HCN2 (siRNA ID s137595, Ambion) was used at 50 nM for 1, 2 or 3 days using Gene Silencer from Genlantis (San Diego, CA).

***Semi-quantitative PCR:*** mRNA was extracted from DRG neuron cultures treated for 1 day with scrambled siRNA (scr) or HCN2 siRNA using RNAeasy kit (Qiagen). Reverse transcription was done using Omniscript (Qiagen) followed by PCR reaction (31 cycles) with Phusion DNA polymerase (Finnzymes, Finland). Primers as follows: for HCN1, 5’-CTC TTG CGT TTA TTA CGC CTT T-3’ (left primer) and 5’-CCT TGG TAT CGG TGC TCA TAG T-3’ (right primer); for HCN2, 5’-CAC TCG TGG AGC GAA CTC TAT T-3’ (left primer) and 5’-CCC TCT CGG ATG ATG TAG TCT C-3’ (right primer) and for GAPDH, 5’-GGT GCT GAG TAT GTC GTG GA-3’ (left primer) and 5’-GGA TGC AGG GAT GAT GTT CT-3’ (right primer). Product size fragments were as expected: 480 bp for HCN1, 532 bp for HCN2 and 360 bp for GAPDH.

***Image analysis:*** Validated scr siRNA conjugated to 5-carboxyfluorescein (FAM; Ambion) was used at 10nM as a negative control and to aid identification of transfected neurons. Staining at the outer limit of the cytoplasm (edge) was examined with image analysis (described fully in the main paper under fluorescence image analysis in cultured neurons).
